# Lynx x-ray microcalorimeter

**DOI:** 10.1117/1.JATIS.5.2.021017

**Published:** 2019-05-31

**Authors:** Simon R. Bandler, James A. Chervenak, Aaron M. Datesman, Archana M. Devasia, Michael DiPirro, Kazuhiro Sakai, Stephen J. Smith, Thomas R. Stevenson, Wonsik Yoon, Douglas Bennett, Benjamin Mates, Daniel Swetz, Joel N. Ullom, Kent D. Irwin, Megan E. Eckart, Enectali Figueroa-Feliciano, Dan McCammon, Kevin Ryu, Jeffrey Olson, Ben Zeiger

**Affiliations:** a NASA Goddard Space Flight Center, Greenbelt, Maryland, United States; b Wyle Information Systems, McLean, Virginia, United States; c CRESST and University of Maryland Department of Physics, Baltimore County, Baltimore, Maryland, United States; d ASRC Federal Space and Defense, Beltsville, Maryland, United States; e National Institute of Standards and Technology, Boulder, Colorado, United States; f Stanford University, Department of Physics, Palo Alto, California, United States; g Lawrence Livermore National Laboratory, California, United States; h Northwestern University, Department of Physics and Astronomy, Evanston, Illinois, United States; i University of Wisconsin, Department of Physics, Madison, Wisconsin, United States; j Massachusetts Institute of Technology Lincoln Laboratory, Advanced Imager Technology Group, Lexington, Massachusetts, United States; k Lockheed Martin Space, Palo Alto, California, United States; l Luxel Corporation, Friday Harbor, Washington, United States

**Keywords:** microcalorimeters, x-ray, Lynx, cryogenics, telescope

## Abstract

Lynx is an x-ray telescope, one of four large satellite mission concepts currently being studied by NASA to be a flagship mission. One of Lynx’s three instruments is an imaging spectrometer called the Lynx x-ray microcalorimeter (LXM), an x-ray microcalorimeter behind an x-ray optic with an angular resolution of 0.5 arc sec and ∼2 m^2^ of area at 1 keV. The LXM will provide unparalleled diagnostics of distant extended structures and, in particular, will allow the detailed study of the role of cosmic feedback in the evolution of the Universe. We discuss the baseline design of LXM and some parallel approaches for some of the key technologies. The baseline sensor technology uses transition-edge sensors, but we also consider an alternative approach using metallic magnetic calorimeters. We discuss the requirements for the instrument, the pixel layout, and the baseline readout design, which uses microwave superconducting quantum interference devices and high-electron mobility transistor amplifiers and the cryogenic cooling requirements and strategy for meeting these requirements. For each of these technologies, we discuss the current technology readiness level and our strategy for advancing them to be ready for flight. We also describe the current system design, including the block diagram, and our estimate for the mass, power, and data rate of the instrument.

## 1 Introduction

### 1.1 Overview

Lynx is an observatory with an x-ray optic with the same focal length of 10 m as Chandra.^1^ It will have a similar angular resolution as Chandra of 0.5 arc sec but will have orders of magnitude more area and greater off-axis angular resolution, allowing for substantially deeper observations to study arc second astrophysical features in crowded regions of the sky.

There are three main scientific goals of Lynx:

- to see the dawn of black holes,
- reveal what drives galaxy formation and evolution, and
- unveil the energetic side of stellar evolution and stellar ecosystem.

The Lynx x-ray microcalorimeter (LXM) is one of three observatory instruments designed to meet the performance requirements for key measurements. It provides high spectral resolution and high spatial resolution nondispersive imaging spectroscopy over a broad energy range (0.2 to 7 keV in its normal mode, up to 15 keV in its high energy range mode). It is composed of a large array of x-ray sensors that determine the energy of individual incident photons by precisely measuring the temperature rise caused by each.^2^ It will meet many different science requirements of Lynx with different subregions of the detector array. Its Main Array will consist of 1 arc sec pixels over a 5 × 5 arc min^2^ field-of-view (FoV), which corresponds to an array of 50 *μ*m pixels extending over an area of 1.5 × 1.5 cm^2^. In the central 1 arc min region, the Enhanced Main Array will consist of 0.5 arc sec pixels corresponding to pixels on a 25-*μ*m pitch. And there will also be a subarray called the Ultra-High-Resolution (UHR) Array, capable of imaging spectroscopy on sources for x-ray energies up to 2 keV, with an energy resolution of 0.3 eV under 1 keV, optimized to have the best energy resolution for the oxygen VII and VIII lines. Similar to other x-ray microcalorimeter instruments, the LXM will incorporate an anticoincident detector to help with discriminate background events. In general, because the LXM pixels are so small, the background will be much less of a problem than for other x-ray microcalorimeter instruments.

With the LXM, we will be able to:

- Use metallicity in galaxy clusters to *z* = 3 as a probe of galaxy formation processes near the peak of cosmic star formation,
- Study plasma physics effects related to energy feedback in galaxy clusters and groups, and
- Study supernova activity in the recent past in the local group galaxies.

The key applications for the Enhanced and Ultra-Hi-Res Arrays are as follows:

- Determine the effects of active galactic nucleus (AGN) energy feedback on the interstellar medium and determine the physical state of gas near the supermassive black hole sphere of influence in nearby galaxies
- Spatially and spectrally resolve starburst-driven winds in low-redshift galaxies, where the gas is expected to have <1 keV temperatures and move with ∼100-km/s velocities.

NASA has supported the development of x-ray microcalorimeters for over two decades now, although the fulfillment of the potential for ground-breaking observations has only just started. The x-ray microcalorimeter instrument on Hitomi demonstrated this with a spectacular high spectral resolution image of the Perseus cluster, revealing the dynamics of the pervasive hot gas.^3^ However, the very unfortunate premature demise in 2015 has left us still at the dawn of such observations, the observatory failure being in no way related to the microcalorimeter instrument. The future missions, XRISM^4^ and Athena,^5^ will have a major impact, enabling understanding in many areas of x-ray astrophysics. However, the limitations in their optics angular resolution and in their corresponding microcalorimeter array pixel and array sizes will still leave numerous key questions unanswered related to the origin and evolution of the universe and a vast area of discovery space untapped.

NASA has also invested heavily in the further development of x-ray optics this decade, and there have been major advances in these key technology areas. A large area sub-arc second x-ray optic now appears to be achievable.^6^ To match this capability, sufficiently small microcalorimeters to allow sub-arc second imaging have been developed.^7^ Arrays with pixel numbers approaching 100,000 pixels, as is needed to maintain a reasonable FoV with such small pixels, appear to be feasible, as will be described in this paper. And sub-eV energy resolution for x-ray energies below 1.5 keV has been demonstrated,^8^ providing a path of an imaging spectrometer with potentially a resolving power of more than 2000 at the all-important oxygen VII and VIII lines. The Lynx mission is the ideal observatory to take advantage of these successful advances because its science requires improved performance/capabilities from both of these technologies.

There are two basic categories of x-ray microcalorimeters that have been under development for a mission such as Lynx. Both of these operate at thermal equilibrium, in which each x-ray produces a temperature rise proportional to *E*/*C*, where *E* is the energy deposited by the x-ray and *C* is the heat capacity of the device. For these devices, the energy resolution is determined by the accuracy with which a very sensitive thermistor can measure a temperature rise on a background of temperature fluctuations caused by the random transport of energy between a body with heat capacity *C*, and the temperature bath to which is connected by a thermal conductance *G*. This is what is commonly known as thermodynamic noise, which scales as $\sqrt{k_{B}T^{2}C}$. For high energy resolution, low temperatures (<0.1 K) are needed to minimize *C* and to minimize these fluctuations. The two types of equilibrium detectors that have been under further development, with the greatest potential of meeting LXM requirements, are transition-edge sensors (TESs)^9^ and metallic magnetic calorimeters (MMCs).^10^ An important version of these types of microcalorimeter that has been under development, making the LXM requirements achievable, are those in which multiple pixels are read out using the same sensor, commonly called Hydras^11^ to be described in Sec. 3.1.1. There has recently been some impressive progress in the development of these.^12^ There has also been impressive progress with a type of multiplexed read-out of microcalorimeters that utilizes SQUIDs in microwave resonator circuits operating at GHz frequencies.^13,14^ It is the combination of this type of read-out and the advancement of Hydras that has led to the breakthrough in capability for future microcalorimeter arrays, such as envisaged for the LXM. LXM will be more capable than any other x-ray microcalorimeter developed to date.

In this paper, we describe the LXM arrays and their requirements and trace them back to key science goals for the Lynx mission. We define technology readiness levels (TRLs) that we believe are appropriate for assessing the readiness of this technological approach for flight. We describe the current state-of-the-art of TES and MMC detector arrays and of the microwave read-out assumed to be used for both of these detector approaches. We describe our preliminary design for the LXM cooling system, both for the cryocooler for cooling down to 4.5 K and the adiabatic demagnetization refrigerator (ADR) for cooling from 4.5 K to 50 mK. And finally, we describe the LXM system design and estimated resources are needed for operating this instrument in space and a plan for developing the critical technologies to be ready for this mission.

### 1.2 Heritage

The preliminary design of the LXM relies heavily on heritage from the x-ray microcalorimeter instrument on Hitomi’s soft x-ray spectrometer (SXS),^15^ from the detailed design and development of the Athena X-ray Integral Field Unit (X-IFU) instrument^5^ and from the sounding rocket that uses a TES x-ray microcalorimeter array called Micro-X.^16^ The block diagram for the LXM will be described in more detail in Sec. 7. The design of the cryostat relies on heritage learned from the SXS regarding the design of the vibration isolation that was necessary to avoid performance degradation from vibrations. We note that the TES technology used by the X-IFU and the LXM is based on a first stage SQUID amplifier with a low impedance input, which is inherently less sensitive to vibrations of the sensor and its wiring. Nevertheless, it is critical to minimize vibrations both from a consideration of the LXM performance and so that the observatory performance is not affected by vibrations when the LXM is not at the focus of the mirror. The LXM includes an assembly with a modulated x-ray source (MXS) that is capable of providing pulsed x-ray lines at multiple energies and is similar to that used on Hitomi’s SXS^17^ for in-flight calibration. Infrared (IR)/optical blocking filters that are necessary to block long-wavelength photons from being incident on the microcalorimeter array are also included and based on the SXS and X-IFU designs. The ADR based for the LXM and its control electronics are based on those used on the SXS, with additional stages of similar design being added to provide additional cooling power.^18,19^ The burst disc, filter wheel, pump-out port, by-pass valve, dewar door mechanism, and event signal processor electronics and software are based upon those developed for and used in the SXS. Since the baseline sensor technology are TESs, there are many advances being made for the X-IFU that will be leveraged for the LXM. In particular, the design of the focal plane assembly (FPA) for housing the sensor array, cold read-out, and anticoincidence detector (utilized to reduce the background x-ray events) for the X-IFU^20^ between 4.5 K and 50 mK is the basis for the LXM FPA. Particular care is needed for shielding magnetic fields for TESs as compared with the silicon thermistor sensors used on the Hitomi SXS.

## 2 LXM Array Design and Science Traceability

**Fig. 1:**
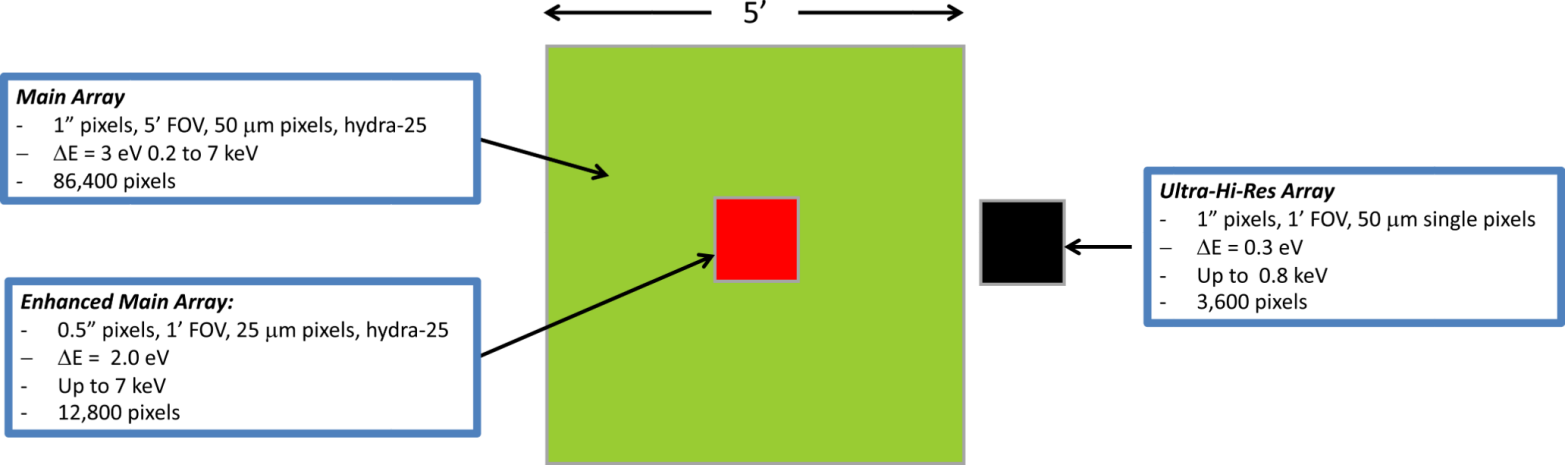
This image shows the baseline layout of the LXM focal plane array. It consists of three different styles of pixels in three different regions. The Main Array consists of 1 arc-sec pixels over a 5 arc-min FoV with a 7-keV energy range, and the Enhanced Main Array 0.5 arc-sec pixels in the innermost central 1 arc-min region. Off to one side is the Ultra-Hi-Res Array consisting of 1 arc-min pixels over a separate 1 arc-min region, optimized for high-energy resolution below 1 keV, but will still have high energy resolution up to 2 keV.

**Fig. 2:**
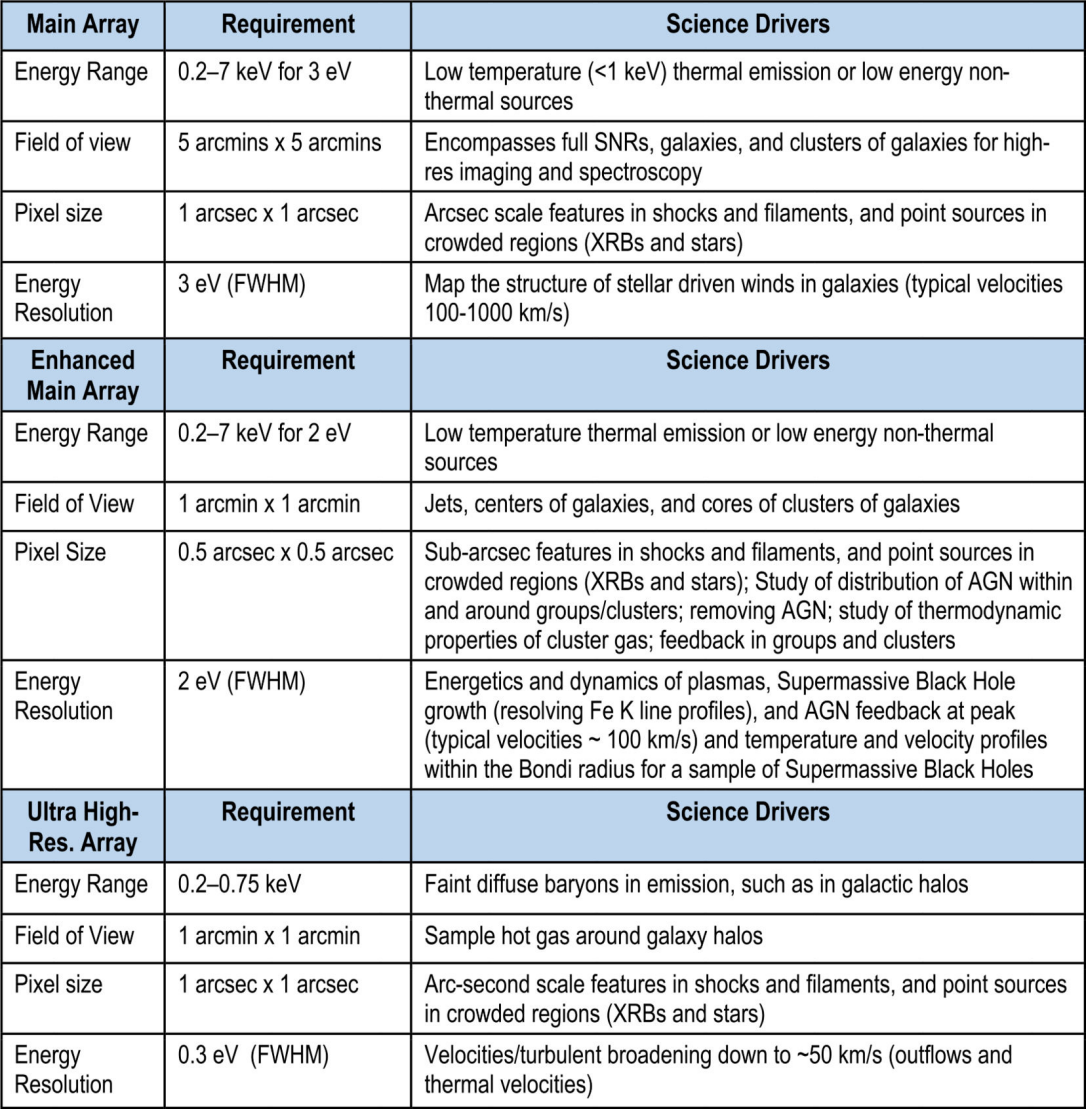
This table summarizes the main requirements of each of the different subarrays, and the main science drivers for these requirements. A thorough list of requirements is given in Sec. 9.

Compared to the planned Athena X-IFU,^21^ the LXM Main Array has the equivalent FoV but with much smaller pixel size, necessary to exploit the x-ray mirror assembly half-power diameter of 0.5 arc sec. This fine resolution will permit Lynx to observe sub-arc second scale features in clusters and jets and minimize source confusion in crowded fields. The layout of the LXM focal plane array is shown in Fig. 1, and a list of the key requirements and their scientific drivers are given in the table in Fig. 2. A more detailed list of requirements is presented in Sec. 9. The baseline configuration utilizes a 5 arc min region consisting of multiabsorber devices known as “Hydras,” to be described in Sec. 3.1.1. In each Hydra, the temperature sensor being read out (TES or MMC) is attached up to twenty-five 1 arc sec absorbers (pixels), 50 *μ*m in size. Discrimination between absorbers from an x-ray event is possible because the different thermal links to each absorber cause characteristic pulse shapes that can be discriminated.

The Main Array will provide an energy resolution of better than 3 eV (FWHM) over the energy range of 0.2 to 7 keV with pixels sizes that vary in scale from 0.5 arc sec in the innermost 1 arc min Enhanced Main Array to pixels that are 1.0 arc sec extending out to a 5 arc min FoV. The Enhanced Main Array will provide 2-eV energy resolution (FWHM) as well as better angular resolution. The pixels and readout are designed to provide this energy resolution up to 7 keV. However, the energy range is extendable up to 15 keV by utilizing a different operating mode that requires no additional instrumentation, in which the temperature of the sensor array is increased to be much closer to the TES transition temperature. This degrades the energy resolution but allows a much larger energy range, as described in Sec. 3.4. Currently, the baseline Lynx energy band does not have an appreciable area up to 15 keV but adding an area in this energy range could be a low-cost enhancement option if the higher-energy response is desired.

A different subarray called the UHR will provide an energy resolution of 0.3 eV up to ∼0.8 keV and < 3 eV resolution up to ∼2 keV (see Sec. 9) in a 1 arc min region off to the side with 1 arc sec pixels. This region will allow the measurement of turbulence in winds of individual galaxies and is also used in a variety of other measurements of hot gases around galaxy halos. The count rate capability is much higher for the UHR array than for the Main and Enhanced Main Arrays and is a derived value based on the intrinsic design of the arrays. The UHR array count rate capability ranges from 80 cps to 1000 cps per 1 arc sec pixel, depending on the required energy resolution (lower resolution allows for higher count-rate capability, see Sec. 9).

An extension of the FoV that is substantially larger than 5 arc min has been considered but is not currently baselined. An extension of 5 arc sec pixels with energy resolution in the range of 1 to 2 eV in an FoV that extended up 20 arc min has been considered. Such an FoV would be ideal for allowing the study of velocities of winds in the outer regions of groups of galaxies. This option is not currently baselined but could be considered during Phase A as an enhanced option for an added cost.

The Main Array requires 3456 TESs, a number similar to the Athena X-IFU, the Enhanced Main Array requires 512 TESs, and the UHR array a further 3600 TESs. In terms of required detector wafer size, number of TESs, and required FPA size, this is within the envelope of what is already considered possible, being similar in scale to the FPA for the Athena X-IFU.

## 3 Microcalorimeter Array Development

### 3.1 Transition-Edge Sensors

**Fig. 3:**
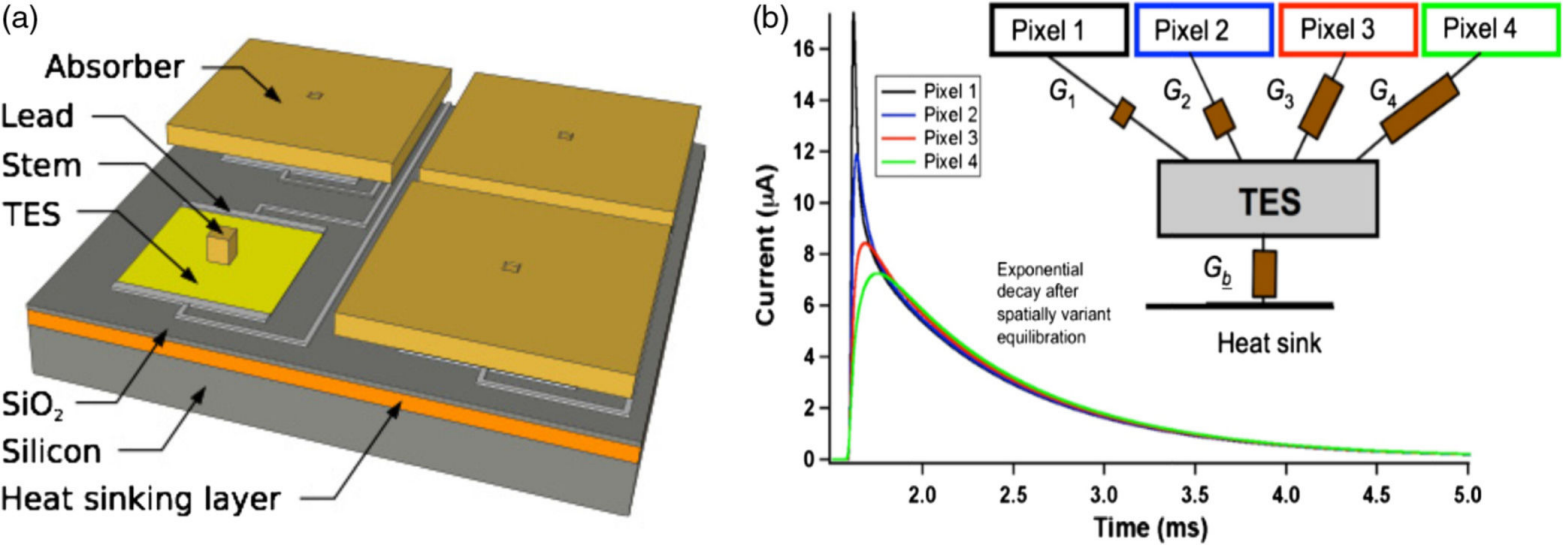
(a) Schematic showing components of a TES calorimeter. This design uses the thermal boundary between the TES film and the substrate for the necessary decoupling of the TES from the substrate. (b) Schematic representation of the TES Hydra. Inset top right: Thermal model of a multipixel TES consisting of four x-ray absorbers, connected to a single TES via varied thermal conductance Gi (where *i* = 1,…,4). The TES is weakly thermally coupled to a heat sink via conductance Gb. The measured average x-ray pulse shapes for a 4-pixel Hydra at an energy of 6 keV are shown. The differences between pulse shapes before equilibration are used to discriminate which pixel the x-ray photon was absorbed in.

Lynx has baselined microcalorimeters with TES thermometers^9^ for this study, single pixel and multipixel Hydra versions, because of the relatively high maturity level compared to other thermometer technologies. The single pixel design is depicted in Fig. 3(a). Multipixel TESs, or Hydras, allow for some degree of thermal multiplexing to reduce the number of TESs that need to be read out, as shown in Fig. 3(b). This multiplexing allows for finer sampling of the FoV as compared with Athena, without a commensurate increase in the number of wires, contacts, and readout components. Large arrays of these sensors are read out using microwave SQUID resonators on high-electron mobility transistor (HEMT) channels. Minimal to no energy resolution degradation from the readout is expected.^22^

#### 3.1.1 Main Array and Enhanced Main Array

Substantial advances have been made in the development of Hydra devices recently, as described in Ref. 12, from this JATIS volume. This includes the demonstration of a Hydra with twenty 50-*μ*m absorbers attached demonstrating 3.36 eV (FWHM) at 5.4 keV, and good pixel event discrimination down to 1.5 keV, and reasonable discrimination at energies as as low as 277 eV. Each pixel in these Hydras was 4.2-*μ*m thick pure gold. Since the energy resolution scales approximately with the square root of heat capacity and thus the thickness of absorber, the energy resolution can be improved to below 3 eV (FWHM) as a trade-off between quantum efficiency at high energies (above 2 keV) and energy resolution.

**Fig. 4:**
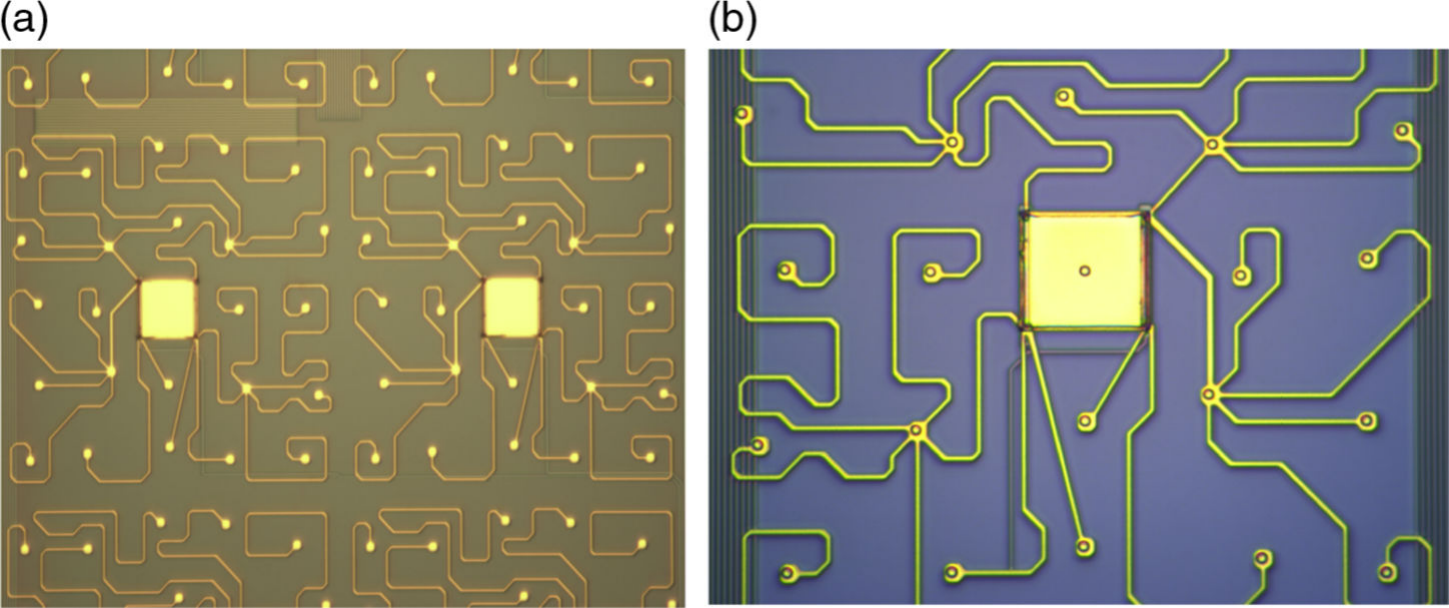
Photographs of prototype LXM Hydras for the Main Array prior to attaching absorbers to the arrays. The TESs are the larger square gold-colored regions and the small gold dots are the locations of the 1.5-*μ*m diameter stems for the different pixel absorbers attached to the TES. The narrow gold-colored lines between the pixels and TESs are the Hydra thermal links, the length of the lines determining the strength of the thermal conductances. The lines are 1-*μ*m wide and 300-nm thick. The pitch of the TESs is 250 *μ*m. (a) This photograph shows two different Hydras in an array. (b) This photograph shows a more zoomed in view of one of the Hydras.

Recently, some Hydras with 25 absorbers attached have been modeled and designed to meet the LXM specifications including some Hydras with pixels on a 25-*μ*m pitch meeting the Enhanced Main Array specifications as well as for the 50-*μ*m pitch Main Array. The preliminary design parameters are shown in Tables 1 and 2. A key part of the design process was to design the TESs and thermal links in such a way as to minimize the read-out requirements. In practice, this means that the maximum slew rate (the rate of change of current signal that needs to be measured from the highest energy photon to be measured) needs to stay as low as possible. This is achieved through a combination of reducing the thermal conductance of all the pixels to the TES (including the ones closest to it) and the addition of some high specific heat capacity metal with strong thermal coupling to the TES. A high specific heat material is desired to keep the thermal conductance to the heat bath as small as possible by keeping the area low. In Figs. 4(a) and 4(b), you can see different thermal links between the TES and the 25 associated absorbers, which are the meandering narrow metal gold films on the surface of the supporting wafer, before the absorbers are added. Because these have only recently been fabricated, they have not yet been tested. It is expected that the Enhanced Main Array pixels will have better energy resolution than the Main Array, ∼2 eV since the heat capacity per Hydra will be 4 times less.

#### 3.1.2 Ultra-Hi-Res Array

The projected performance of the Ultra-Hi-Res Array is based upon the detector described by Lee et al.^8^ In this paper, a single microcalorimeter pixel was described that achieved an energy resolution of 0.72 eV (FWHM) at 1.5 keV in pixels that were on a pitch of 50 *μ*m, with all-gold absorbers that were 4.2-*μ*m thick. For this device, the predicted energy resolution at lower energies was 0.5 eV although no calibration source was available to verify this. Based on the characterization of this device, it was shown that due to some contamination in the gold, the specific heat capacity was 53% higher than predicted for elemental gold. By modeling, Lee was able to show that ∼0.4 eV should have been possible if the heat capacity had been as expected for gold without any magnetic impurities.

As the thickness of the absorber decreases, so does its heat capacity and usable energy range, roughly in proportion. Thus, there is a trade-off between energy resolution and energy range in order to achieve the best possible energy resolution for a given energy. For the UHR, it was decided that the oxygen lines around 0.6 keV were the most important line energies (O VII triplet: 574 eV, 569 eV, 561 eV, O VIII Ly*α*: 654 eV) as well as the first two strong lines Fe L lines near 717 and 733 eV. Based upon this, we modeled a design of pixel with a thinner absorber, 2-*μ*m thick instead of 4.2 *μ*m, and estimated that an energy resolution of roughly 0.3 eV is possible at low energies. For energies higher than this, the response will be nonlinear, and the resolution will degrade slowly toward around 3 eV at 2 keV. Some signal processing algorithms that involve the studies of nonstationary noise in nonlinear detectors could potentially improve the resolution between 1 and 2 keV^23^ to significantly less than 2 eV, but the use of these is not baselined. The requirement for 0.3 eV resolution, therefore, extends only to a maximum energy of 0.8 keV. These pixel design parameters are given in Tables 1 and 2.

### 3.2 Progress in the Fabrication of Large LXM-Style Arrays

The requirements for the LXM array are challenging as they represent such a dramatic increase in array size compared with any microcalorimeter array envisaged previously, and because it requires pixels to be on such a fine pitch. In particular, it is challenging in two key areas. The first is in the capability to read out of this large number of pixels with a multiplexed read-out, which will be addressed in Sec. 4. The second is the routing of a very large number of high-density superconducting microstrip wires from the pixels to the read-out. This needs to be achieved with very high yield and with very low electrical cross-talk. In both the Main Array and the Enhanced Main Array, under the assumption that wires need to be routed in a planar fashion, the gaps between the outer pixels of two neighboring Hydras are in the range of 25 (for the Enhanced Main Array) to 50 *μ*m (for the Main Array). In these arrays, as many as 34 separate microstrip wires need to pass between neighboring pixels. While microstrip with pitches on the submicron scale is conceivable, this would be a massive technical challenge. One can conceive of geometries in which signals are passed through the wafer vertically onto a carrier wafer before the wires fan out to an underlying readout, such as has been demonstrated for some TES bolometer arrays.^24^ However, the much smaller pixel pitches and the need for much greater pixel heat-sinking make such an approach impractical for this application. This is because a heat bath consisting of a continuous highly conductive metal film is needed directly below the wiring and pixels in order to minimize thermal cross-talk between pixels.^25^

Based upon the high density of wires needed and the challenging constraints on the wiring, the use of buried layers of multilayer wiring is the most attractive way to meet the wiring requirements. A promising multilayer Nb process has been under development by a few places, such as Hypres and Massachusetts Institute of Technology Lincoln Laboratory (MIT/LL).^26–28^ MIT/LL recently developed a process that supports eight superconducting Nb metal layers for superconducting electronics. It utilizes extreme UV (EUV) lithography to achieve submicron line/space resolution and chemical mechanical polishing (CMP) to ensure all metal layers are deposited on a surface with topography height of less than 40 nm. Extremely high yield has been demonstrated in these processes for submicron Nb linewidths. Using this process, multilayer buried wiring can lower the cross-talk. By surrounding the signal lines with grounded shielding layer, cross-talk can be reduced by a large factor. Even greater electrical isolation as large as −65 dB can be achieved on two strip lines that are 1 *μ*m apart using a stacked via a wall of ground planes between two signal lines. This process has the major benefit of producing a planarized surface on top of the wiring to fabricate the main microcalorimeter pixels with generally much more space for the pixel designs than other wiring approaches. The recently formed collaboration between GSFC and MIT/LL leverages over a decade of substantial investment in infrastructure and process development at MIT/LL for superconducting digital electronics and quantum computing.

**Fig. 5:**
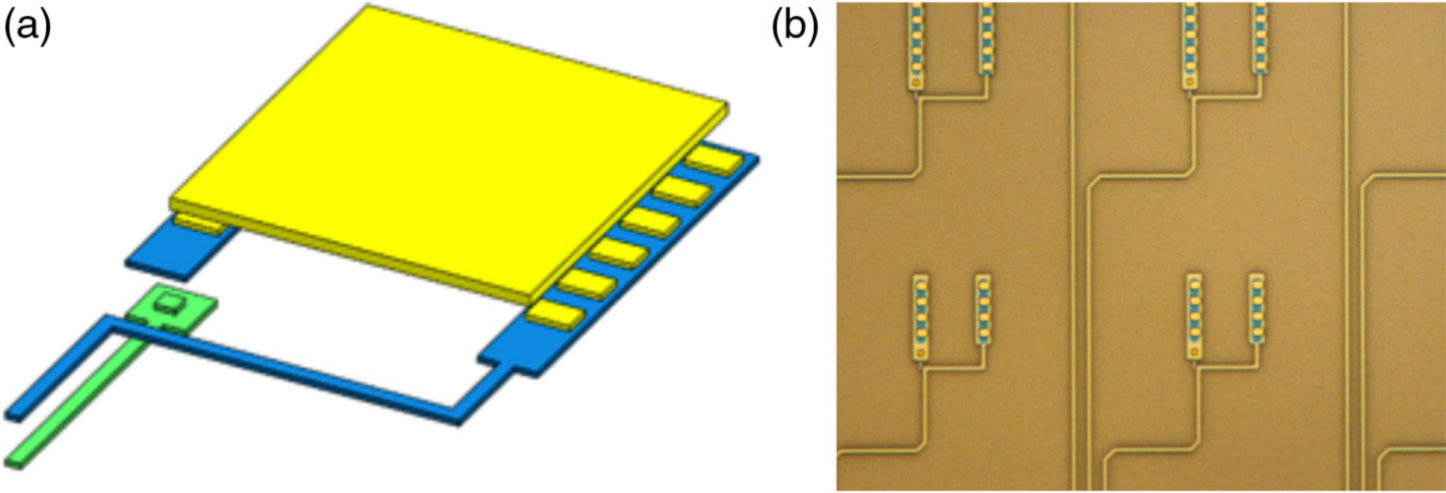
(a) An exploded cartoon of the second lead attachment process (with two sets of vias through the oxide) showing how the wiring mates to the GSFC TES fabrication process. (b) Photograph of a chip with an attachment between buried superconducting wiring and a TES through a process in which Nb oxide is removed from MIT/LL wires, where the surface of the top layer Nb is planarized.

A collaboration between GSFC and MIT/LL has demonstrated that the fabrication of buried multilayer superconducting wiring and the fabrication of TES arrays can be successfully integrated. The first TES microcalorimeter arrays fabricated with buried wiring are shown in Fig. 5. Measurements indicated that the yield of the wiring was 100%. This included tests of the superconductivity of all Nb layers of different widths and all superconducting vias between layers. The measured critical current is uniform, around 30 mA across all devices at 4.2 K, which is what is expected for the 500-nm linewidths used and the 1-*μ*m vias between layers. A number of pixels in the array were tested to demonstrate their performance as x-ray microcalorimeters. The only suboptimal aspect of the first arrays yielded was that the transition temperature of the arrays was around 170 mK (temperature of bilayer under optimum bias conditions), instead of around 65 mK as would be desired for the LXM array. The superconducting transitions were smooth and have shapes and properties as expected based upon measurements of previous arrays using the standard GSFC process. The spectral performance of the pixels was 1.1 eV (FWHM) for Al K*α* x-rays and 1.94 eV for Mn K*α* x-rays.^7^ The Mn K*α* resolution was a little worse due to the nonlinearity of the response. By reducing the transition temperature to 65 mK, the energy resolution could improve to ∼0.3 eV as required by the UHR array. The control of the transition temperature in small TESs such as this is complicated by the presence and reproducibility of the lateral proximity effect from the Nb leads attached to the TES.^29^ This issue has been successfully addressed in more recent devices through the addition of a TiAu gold layer in-between, now providing more reproducible proximity effects.

**Fig. 6:**
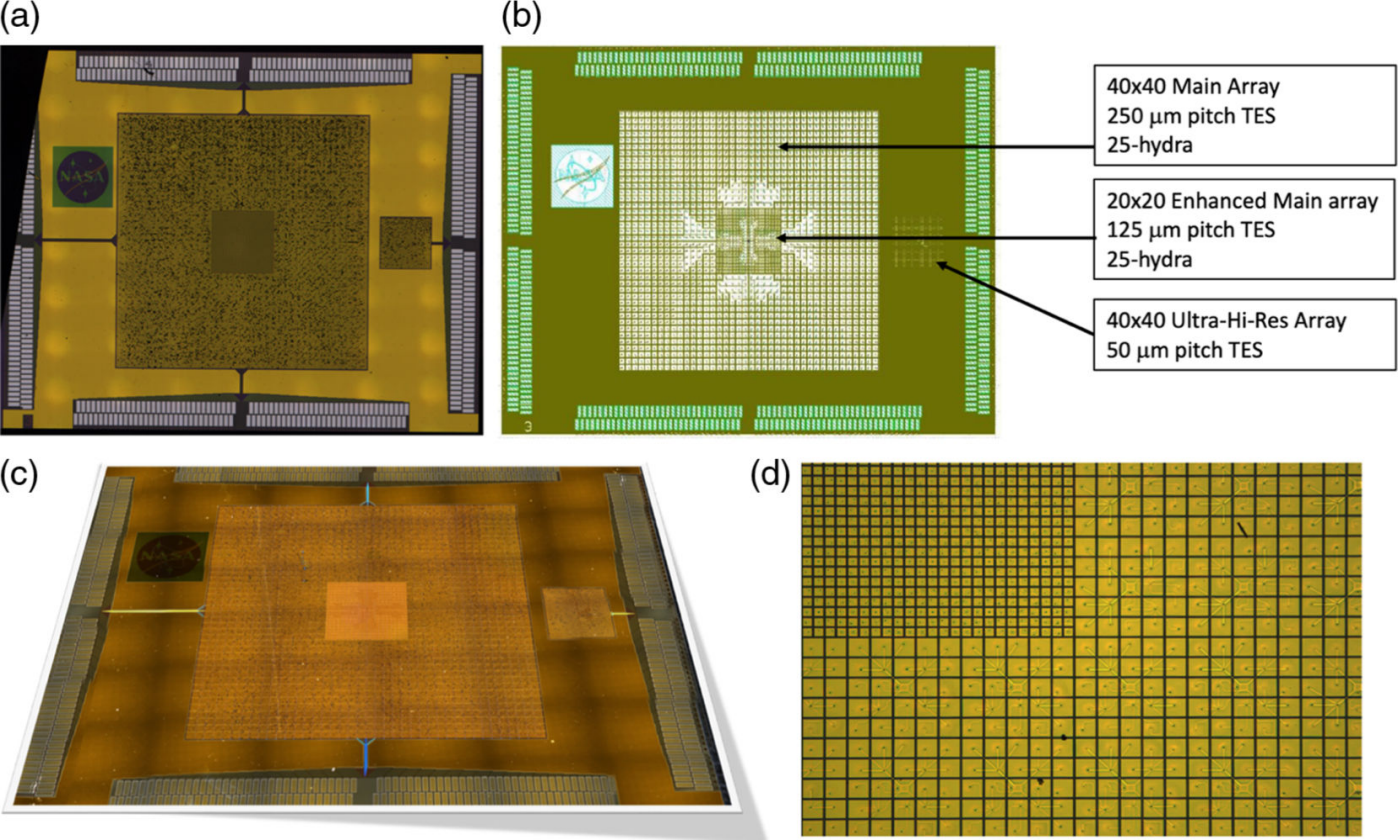
Prototype LXM arrays. The dimensions of this chip are 1.9 cm × 1.5 cm. (a) Photograph of an overhead view of a prototype LXM array with nearly 50,000 pixels. (b) Mask layout showing different regions of the array and the internal wiring for a subset of the pixels. The labels show the regions and number and pitches of pixels for the three different subarrays. (c) Angled photograph of the completed entire array. (d) Zoomed-in view of a region of the array showing the interface between the Main Array and the Enhanced Main Array.

**Fig. 7:**
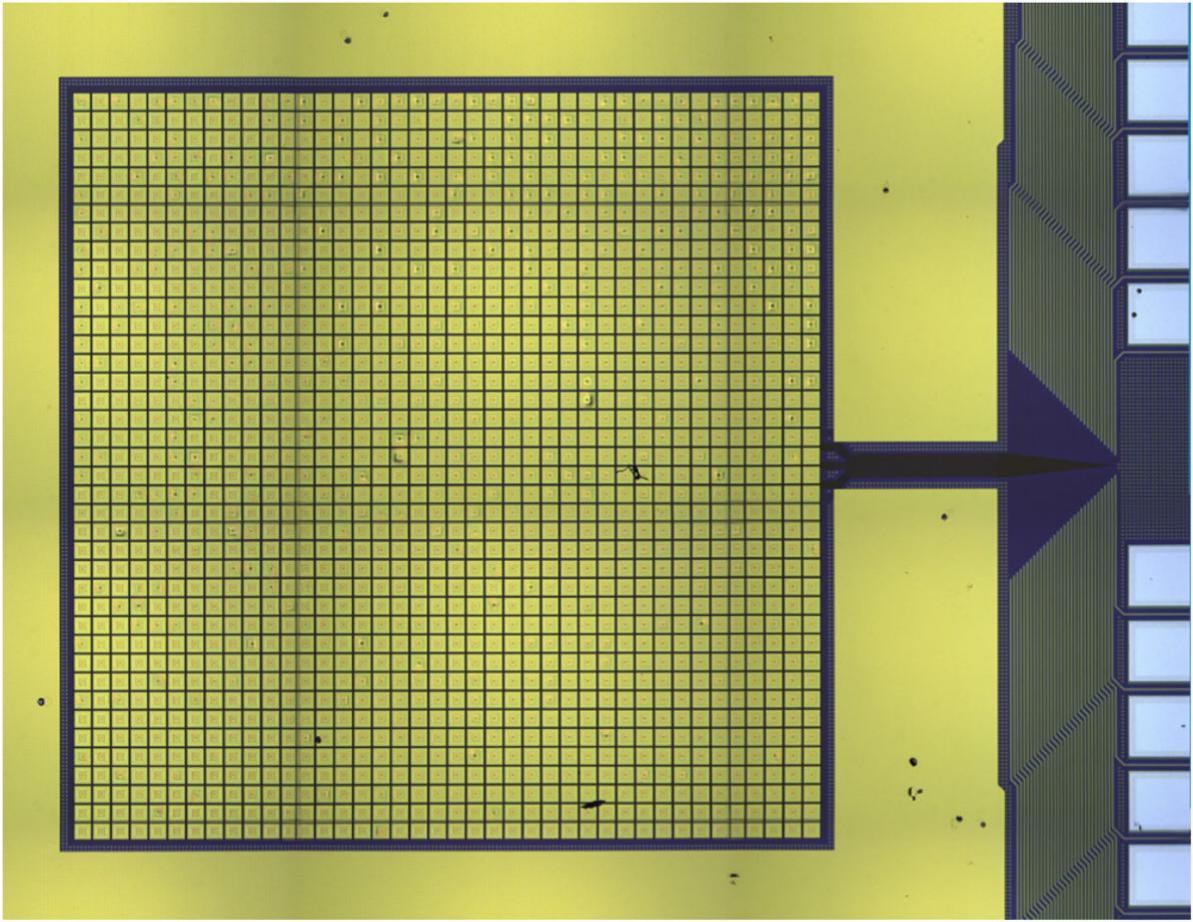
Photograph of the prototype UHR array. This is a 40 × 40 array of single pixels on a 50-*μ*m pitch. Each absorber is 1-*μ*m thick.

The latest prototype LXM detector arrays that have been fabricated are shown in Fig. 6. The Main Array and Ultra-Hi-Res Array are both two-thirds size of the full-scale LXM baseline in linear size. The Enhanced Main Array shown is 83% of the full-scale array. The Main Array consists of 37.5 kilo-pixels, the Enhanced Main Array has 10 kilo-pixels, and the UHR has 1.6 kilo-pixels for a total of 49.1 kilo-pixels, about a half of the total for the full-scale array. A photograph of a zoomed-in image of the UHR array is shown in Fig. 7. This prototype incorporates buried superconducting microstrip wiring to a subset of the pixels in the array, including pixels from each of the three subarrays, with these wires fanning out to the 256 pairs of wirebond-pads around the perimeter of the chip. The layout and dimensions of the wiring are consistent with being able to wire out every pixel in all three of the subarrays, but full-scale wiring was not included in these first TES arrays. Currently, the Main Array and Enhanced Main Array pixels have not been tested. Early test results from some UHR pixels from the first array to yield are given in Sec. 3.3. Results from the thorough characterization of all these pixel types are expected in early 2019.

The prototype array shown in Fig. 6 has 1-*μ*m thick absorbers everywhere. Arrays have been produced for this scale of pixel absorber size for thicknesses that range from 1 to 4 *μ*m.^7^ At GSFC, we will also be fabricating versions, which have different absorber thicknesses for the different regions of the array, using fabrication techniques previously demonstrated.^30^ Approximately 4 *μ*m of Au is desirable to have high quantum efficiency up to 7 keV for the Main and Enhanced Main Array. About 1 to 2 *μ*m of Au is desired for the UHR Array. The fill factor for each pixel will depend upon the gaps between absorbers. In general, how narrow this gap can be depends on the thickness of absorber. In our first prototype arrays, the gaps between absorbers is 4 *μ*m, for 4-*μ*m thick Au absorbers. Optimization of the ion-milling process should allow this gap to be reduced below 2 *μ*m. We have already demonstrated such small gaps in 1-*μ*m thick absorbers. Gaps less than 2.6 *μ*m are necessary to meet the current fill-factor requirement, 90% for the Main Array and 80% for the Enhanced Main Array.

### 3.3 Preliminary Test Results on the UHR Array

**Fig. 8:**
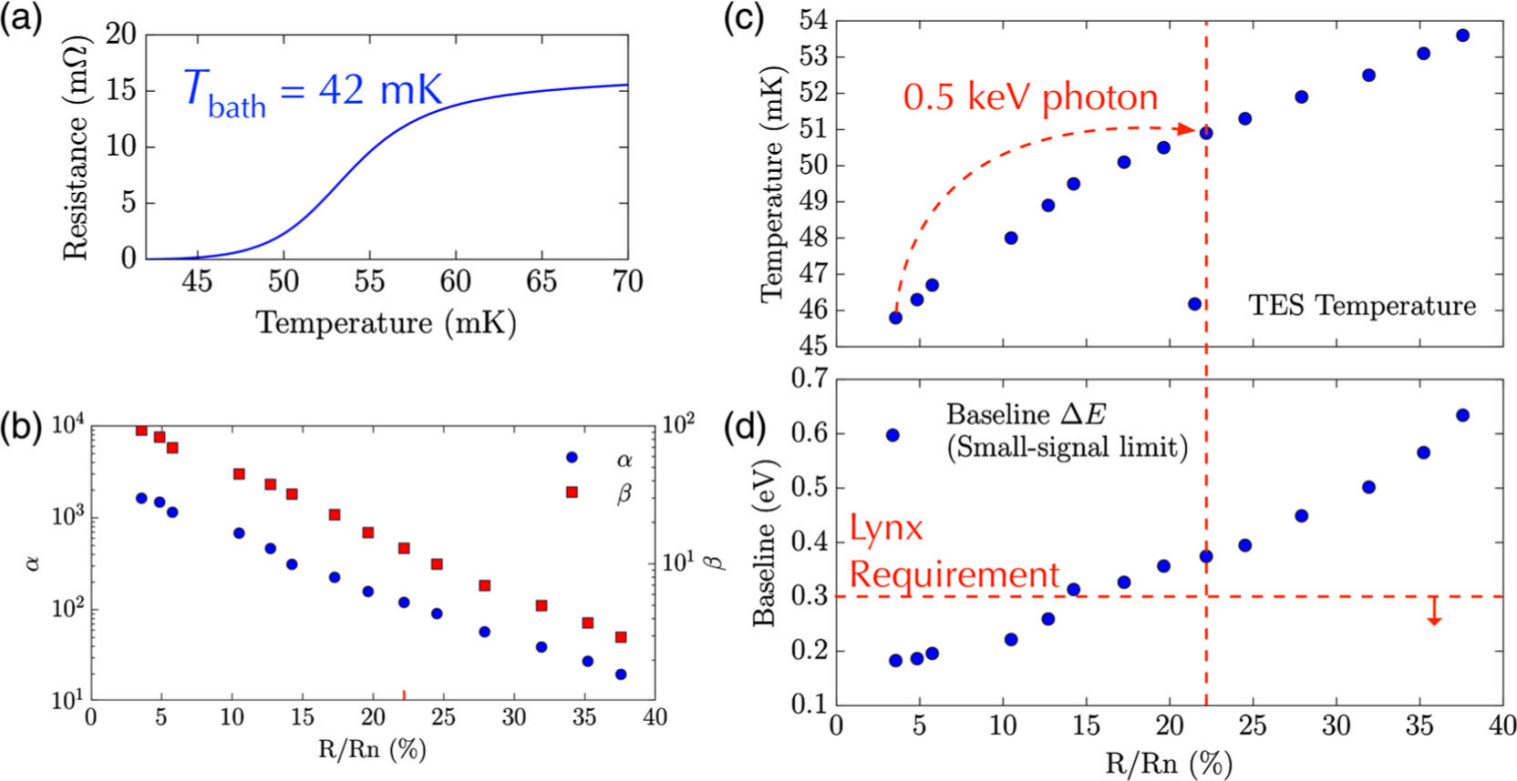
Results from the UHR array fabricated. (a) Measured resistance versus temperature curve. (b) Measured *α* and *β* for this pixel type as a function of the TES bias level (as a percent of the normal resistance), as determined from impedance measurements. (c) Temperature as a function of TES bias level. (d) Measured baseline resolution as a function of TES bias level, as determined by the TES responsivity (*α*/*β* from impedance measurements) and the measured noise at the corresponding bias points.

**Fig. 9:**
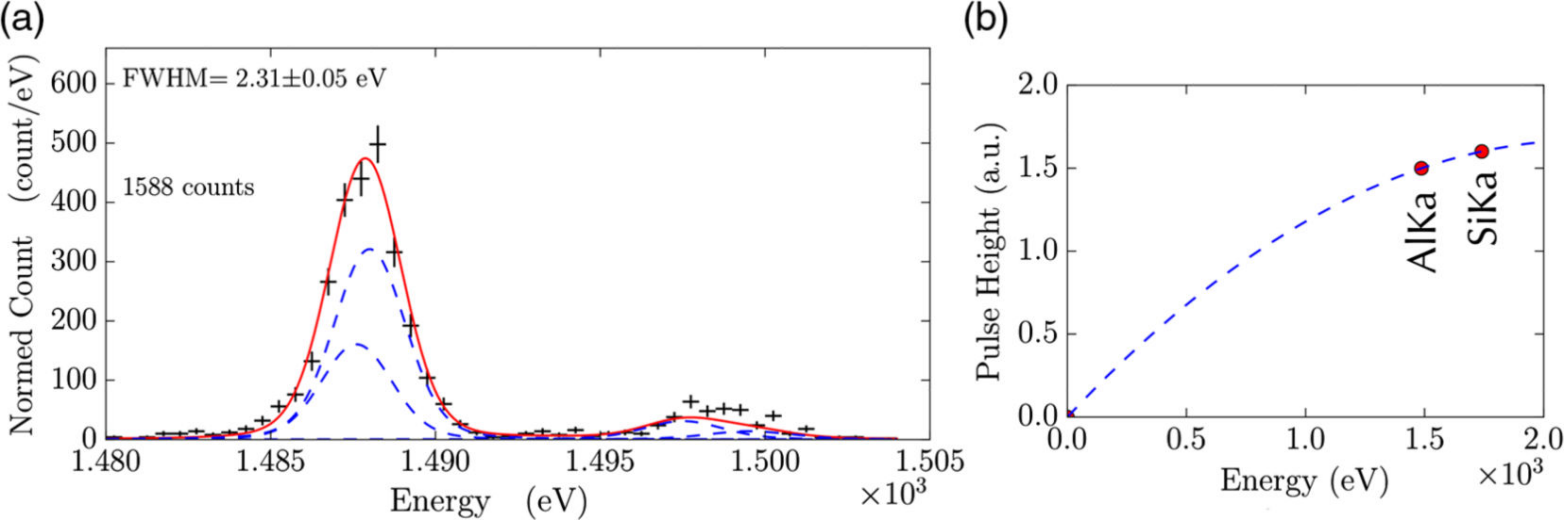
(a) Measured spectrum in prototype UHR array for Al K_*α*_ x-rays. The red line is a best fit through the data corresponding to a FWHM broadening on the Al K_*α*_ x-rays of 2.31 ± 0.05 eV. The dashed blue lines represents the four broadened Al K_*α*_ x-rays lines broadened by this same detector resolution. (b) Filtered pulse height as a function of energy for the observe Al and Si K_*α*_ x-rays.

We have carried out our first tests on a UHR array of the type shown in Fig. 7. The transition temperature under bias was about 10 mK lower than the target of 65 mK, and so, this array need to be characterized with a thermal bath temperature of 42 mK instead of 50 mK. The resistance versus temperature curve is shown in Fig. 8(a). We have not yet been able to measure the energy resolution of these detectors with x-rays in the energy range of 0.2 to 0.75 keV. However, we did use the array to measure Al K_*α*_ x-rays, and the spectrum is shown in Fig. 9(a). The FWHM energy resolution was 2.3 eV at 1.5 keV, demonstrating that we can use this array to achieve excellent energy resolution well above the energy range where it is designed to achieve its best energy resolution, where the response becomes very nonlinear, as can be seen in Fig. 9(b). The temperature rise we expect for the 1.5 keV x-rays is ∼15 mK, and from Fig. 8(a), we can see that these energy x-rays will cause the TES to go into a region of the TES transition that has relatively low responsivity.

In order to estimate the performance for lower energy x-rays, we measured the impedance and noise spectrum for a variety of points in the superconducting transition^31^ to determine *α* and *β*. ($\alpha =\frac{\partial \;\log\;R}{\partial \;\log\;T}$ and $\beta =\frac{\partial \;\log\;R}{\partial \;\log\;I}$, where *R* is the TES resistance, *T* is the TES temperature, and *I* is the TES current). *α* and *β* are plotted as a function of the bias point in Fig. 8(b). From these measurements, we can make good estimates of the “baseline” resolution at low energies, and these are plotted as a function of the TES bias point in Fig. 8(d).

As can be seen in this plot, the estimated energy resolution for low energies at the lowest bias points is below 0.2 eV. However, as we bias higher up the superconducting transition, the estimated small-signal energy resolution increases, crossing the Lynx requirement of 0.3 eV energy resolution around 15% of the normal resistance. It is hard to estimate precisely the energy resolution when an x-ray traverses a region of the resistance–temperature–current surface during an x-ray event. However, it is typically between the small-signal resolution at the initial bias point and small-signal resolution that corresponds to the expected resistance at the maximum temperature during an x-ray event. We estimate that a 0.5-keV photon will produce a temperature rise of ∼5 mK. Therefore, a 0.5-keV photon will likely cause the resistance to increase up to ∼22% of the transition. The expected energy resolution for x-ray energies between 0.2 and 0.5 keV is between 0.18 and 0.35 eV. We, therefore, estimate that the energy resolution will be under the ∼0.3 eV requirement for the UHR array for x-rays up to 0.5 keV, but the exact solution will depend on details of the nonstationary noise during the pulse. We will test the energy resolution of these pixels as a function of energy in detail in the near future using pulses of a large number of 3 eV photons from a laser diode^32^ calibration source. The key parameter that we can adjust in this design to vary the energy range and energy resolution is the heat capacity of the absorber. As the heat capacity is increased, the small-signal energy resolution becomes slightly worse. However, the slope of resolution versus energy excursion, such as is shown in Fig. 9(b), becomes less steep, providing a broader energy range of x-ray that should remain below 0.3 eV. This type of optimization should allow us to achieve approximately the desired energy resolution of 0.3 eV up to 0.75 keV, as is the current requirement.

### 3.4 Extended Energy Range Modes

The science case for extending the energy range of the Main Array and Enhanced Main Array beyond 7 to 12 keV or even ∼15 to 20 keV if the optic provides sufficient area is broad. It includes improving black hole spin measurements,^33^ expanding our understanding of the x-ray emitting corona associated with accreting black holes, improved measurements of feedback,^34^ studies of obscured AGN,^35,36^ x-ray reverberation mapping to uncover the geometry of the central engine,^37^ and studies of ultraluminous x-ray sources.^38^ The LXM is ideally suited for this since a higher energy response can be achieved without modification to the current instrument design. (A modification to the x-ray mirror via additional mirrors or a multilayer coating would be required. Additional considerations such as the increased mirror manufacturing and calibration would also need to be examined.)

**Fig. 10:**
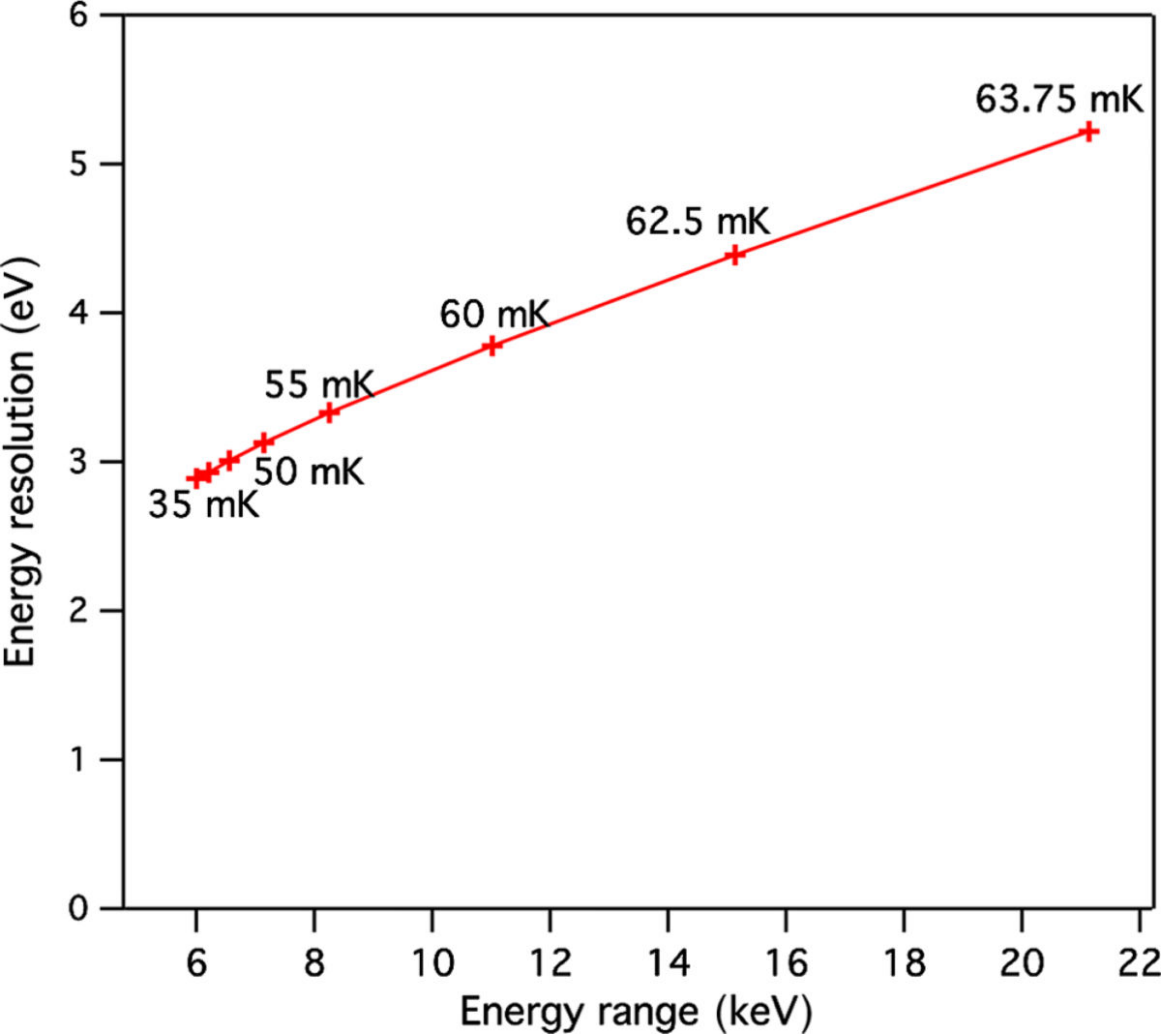
This plot shows how the modeled energy range and energy resolution change as a function of bath temperature as this temperature is increased closer to the superconducting transition temperature for a typical TES x-ray microcalorimeter. The transition temperature, in this case, is 65 mK. This modeled example assumed that the maximum allowable slew rate is constant and sufficient for a 7-keV range and 3-eV energy resolution for a bath temperature of 45 mK. It also assumes that the energy range is limited by the slew rate. In order to extend the energy range above 15 keV, the bath temperature needs to be biased just a few mK below the transition temperature, where the energy resolution is expected to degrade to ∼5 eV.

The energy range of our pixel designs and performance at higher energies will depend on two key factors. The first is the width and shape of the TES superconducting transition, and the second is the slew rate of the multiplexed read-out. In practice, if we accept some significant energy resolution degradation, the biggest constrain in terms of energy range will be due to the maximum read-out slew rate. Figure 10 provides an estimate from modeling for how the energy resolution of the Main Array pixel might change as a function of the operating bath temperature, as this temperature is increased closer to the transition temperature, assumed here to be 65 mK. As can be seen in this figure, by raising the bath temperature close to the transition temperature and setting the TES biases to bias levels to compensate, it is possible to extend the energy range from around 7 keV up ∼15 to 20 keV. The expected degradation from raising the bath temperature would be to around the 5 eV level. Comprehensive measurements are needed to verify the extension of the energy range and the level of energy resolution degradation with bath temperature, but it is certain that the energy range can be extended by operating in this mode. This will only require some additional calibration effort but requires no additional hardware.

### 3.5 Defocusing Mode

There are several types of high count-rate sources that are interesting to target with Lynx. Although they do not necessarily require the spatial resolution made possible by the Lynx optics, the spectroscopic capabilities nevertheless enable avenues of investigations. In addition, the vast majority of bright x-ray sources are time-variable and require time-sensitive observations. The brightest x-ray sources are accreting stellar mass black holes and neutron stars, which can reach fluxes of several Crab (C). For example, Sco X-1 is ∼3 C, as are a number of neutron stars during x-ray bursts. Winds and outflows from accreting sources also reach fractions of a Crab. We know that incisive diagnostics of physical processes and conditions in astrophysical plasmas can be inferred from high-resolution spectra. The target energy resolution is set by the requirement to be able to separate nearby atomic lines, including doublets, and resolve the spectral features in high-velocity winds. The x-ray spectra from iron L-shell ions are particularly important here since they fall in the soft x-ray band below 2 keV. For example, highly charged Fe ions, such as Li- or B-like Fe, show strong features in the 0.2- to 2-keV range. As a specific example, Belike Fe is an excellent density diagnostic and has a separation of 0.01 keV at 1.03 keV (wavelengths of 11.77 and 11.92 Å), which requires a resolving power of 100. Other spectral features can be separated by as little as ∼1 eV, requiring resolving powers of *R* ∼ 1000.

There are two ways in which bright sources can be observed with the LXM. Both of these approaches rely on defocusing and spreading the PSF over ∼1 arc min. This can be achieved by moving the focal plane toward or away from the mirror by ±1 cm, which is already part of the planned capabilities for the LXM. While defocusing naturally sacrifices angular resolution, the bright sources of interest are typically point sources, for which the angular resolution does not play a key role. The UHR array can handle 80 counts/s/pixel in the highest energy resolution mode, which can achieve a 0.3-eV energy resolution. This is estimated to decline to ∼2 eV in the low-resolution mode, predominantly for count rates up to ∼1000 cps, the equivalent of 10 mC/pixel. In between these limits, a medium resolution of ≈1 eV can be achieved for count rates up to ∼320 cps/pixel. With off-axis pointing and defocusing, the PSF of the beam covers ≈1000 pixels. Therefore, a flux of ≈10 C can be observed with lower energy resolution (low-res events, with shorter record lengths, see Sec. 9) and a resolution of 1 eV can be maintained for fluxes up to ≈3 C. This approach will be best suited for relatively soft targets or for features of interest in the soft band.

The Enhanced Main Array can be used for sources that have harder spectra and moderate fluxes, also using defocusing. In this array, 0.1 to 0.2 mC/Hydra can be observed with an energy resolution of ≈2 eV and 150 to 300 cps (or 1.5 to 3 mC) can be accommodated as low-res events (energy resolution <10 eV). A Hydra consisting of 5 × 5 array of pixels covers 2.5″; a 1′ PSF will be spread over 576 Hydras. Sources with Fx ∼1.5 C can, therefore, be observed as low-res events. The main advantage of the Enhanced Main Array is its wider energy range. When spectroscopic features of interest lie above 1 keV for bright sources, this observing configuration will provide an excellent option with *R* ∼ few hundreds to 1000 resolving power.

### 3.6 Current Technical Readiness Level

Our self-assessment of the TRL of the detectors is TRL-3 and is close to TRL-4. Section 9 describes our definitions of TRL’s for TRL-3 through TRL-6 and also breaks down the overall TRL into subcomponent demonstrations for critical technologies and necessary model predictions that LXM requirements can be met. If the recently yielded array/subarrays perform as predicted and meet the performance requirements stated, then we will be able to assert a TRL-4. Most of the effort toward developing detectors for the LXM is taking place at GSFC and MIT/LL supported by NASA. There is a clear plan and schedule of activities to reach TRL-5, as summarized in Sec. 7.3, by 2024.

### 3.7 Magnetic Calorimeters

**Fig. 11:**
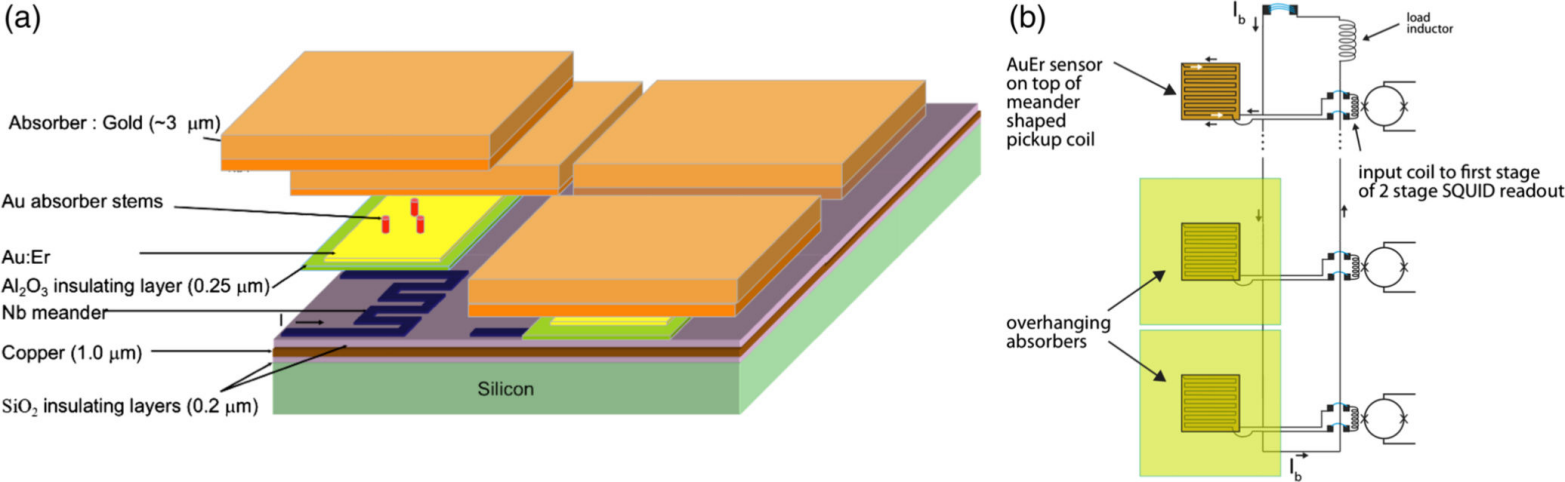
(a) Schematic showing components of an MMC calorimeter. Like the TES, this design uses the thermal boundary between the MMC AuEr sensor film and the substrate for thermally decoupling of the MMC from the substrate. (b) Schematic of the basic circuit for reading out MMCs.

MMCs are an alternative detector technology that is being developed in parallel to TESs that also show great potential for meeting LXM requirements. The operating principle is conceptually simple. When a photon is incident on the absorber, the photon’s energy is converted into thermal energy causing the absorber to warm; it subsequently cools as heat flows out of the absorber and into the heat sink. MMCs utilize the temperature dependence of a paramagnetic material in a weak magnetic field to detect the temperature rise resulting from the absorption of an x-ray. Gold doped with a small amount of erbium (Au:Er) is an effective sensor material. The temperature rise from an absorbed x-ray causes a change of magnetization (*M* ∝ 1/*T*) of the Er spins. When the magnetization of the sensor changes, the resulting change in magnetic flux within the meander pick-up coil generates a change in current through the input coil of a highly sensitive SQUID. The way the MMCs are coupled to a SQUID read-out is shown in Fig. 11(b). A weak thermal link to a heat bath provides a means for the temperature of the MMC to return to its base temperature.

More details on how these detectors work and how they have been adapted to meet the requirements of the LXM are described in detail in Ref. 39 as part of this special section of JATIS. One of the attractive features of MMCs as a parallel detector option is that it uses essentially the same sort of multiplexed read-out being baselined for TESs, namely it uses multiple microwave SQUID resonators operating at GHz frequencies on a single read-out chain through a low-temperature HEMT. It also utilizes some of the same fabrication technologies as TESs. The MMC absorbers are also fabricated with electroplated gold, very similar to those fabricated for TESs. They also make contact with the sensor in a small area. An MMC Hydra is essentially the same as the TES Hydra, except that an MMC sensor replaces the TES. MMCs can also take advantage of the tremendous benefits available from having buried superconducting wiring. In fact, arguably, the buried wiring is even more valuable to MMCs than TESs.

**Fig. 12:**
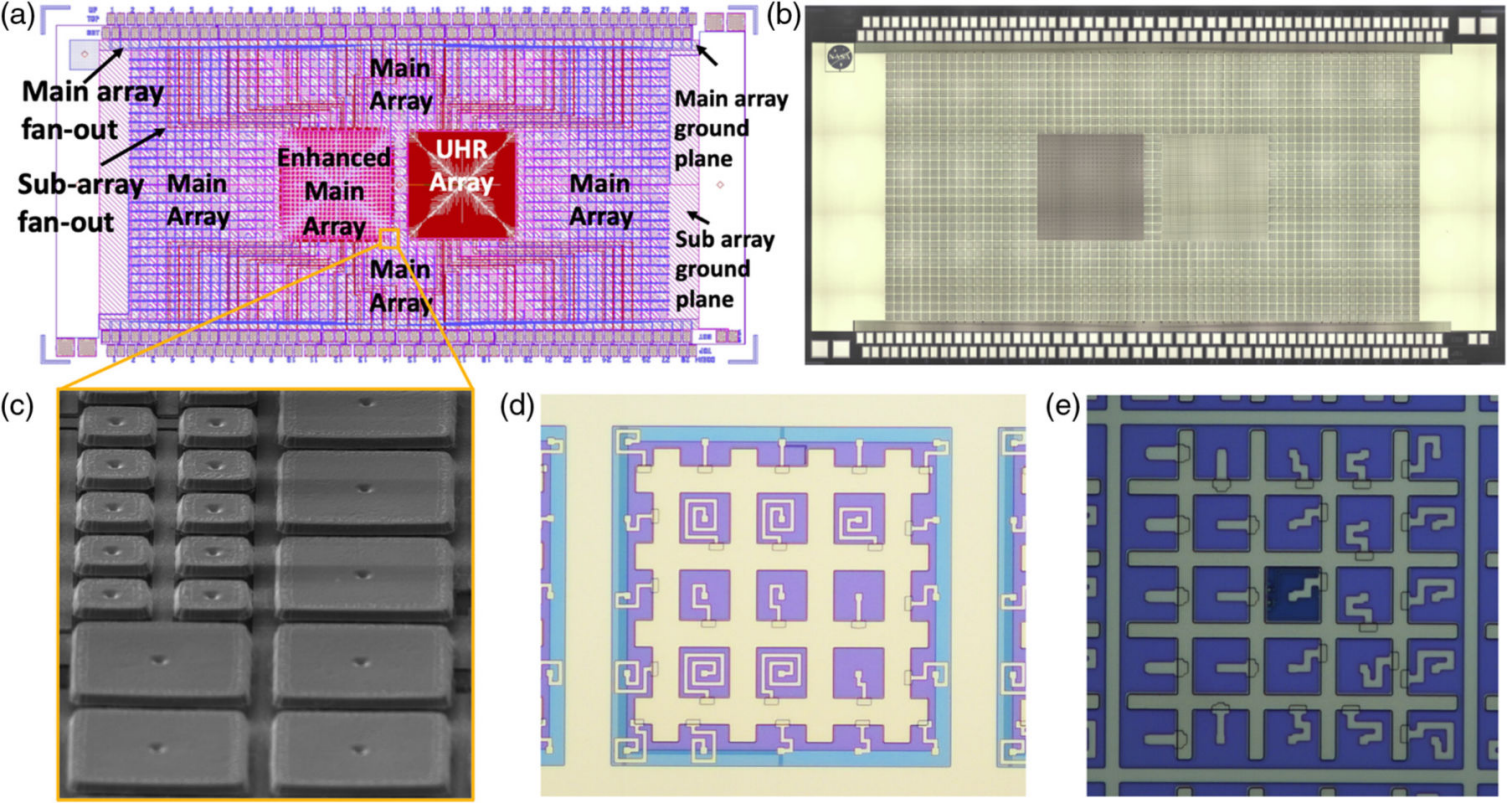
Prototype LXM array using MMCs. (a) Design layout of the entire prototype array in which all of the pixels are wired within the array. The blue lines are the wiring to the Main Array, the purple lines are for the Enhanced Main Array and the red lines are for the UHR array. There are 112 pairs bond pads spread across two rows in along both the top and bottom edges. (b) Photograph of a completely fabricated prototype. (c) Scanning electron microscope image of a small region of the array that shows the interface between the Main Array pixels and the Enhanced Main Array pixels. The 2.8-*μ*m-thick gold pixel absorbers are cantilevered above the substrate on small stems that make contact in the pixel centers, where the pixel surfaces are recessed. The gaps between pixels in this first prototype are 5 *μ*m, designed conservatively to ensure high yield. (d) Photograph of Main Array Hydra before the 25-pixel absorbers was added (pixels on a 50-*μ*m pitch). The raffle-shaped region is the MMC sensor, and the lengths and widths of the lines between the waffle and central pixel stem locations determine the thermal conductance to each pixel. (e) Photograph of an Enhanced Main Array Hydra before the 25 absorbers was attached, these pixels being on a 25-*μ*m pitch.

As part of the collaboration between GSFC and MIT/LL, a prototype LXM array has also recently been produced, which is similar in scale to the version developed for TESs. This prototype is shown in Fig. 12.

This prototype chip is 2.0 cm × 1.0 cm and contains bond-pads for reading out a sample of 56 MMC sensors on each side, for a total of 112 sensors (single pixels and Hydras) available for testing. The layout is slightly different in approach to the TES LXM prototype. It consists of a basic envelope that is half of the LXM Main Array in scope, with 30 rows of MMC sensors and 60 columns, with each sensor connected to 25 pixels in a Hydra geometry. Within this envelope are full-size prototypes of the other two subarrays. There is a full-size Enhanced Main Array in the left central region, with 24 × 24 arrays of 5 × 5 Hydras. Then, there is a full-size UHR 60 × 60 array with single 50-*μ*m pixels. In total, there are 55.8 kilo-pixels in this array. What is even more impressive is that every single pixel is wired from the pixels to the outside of the region of the array. This was achieved using an automated algorithm for laying out the positions of all the wires. It required the use of four different Nb layers, as opposed to the two layers used in the TES prototype. See Ref. 39 for more details.

MMCs have several key advantages over TESs but are currently at a lower state of development. The main advantages are as follows: (1) Theoretically, MMCs have the potential for even greater energy resolution than TESs because there is no Johnson noise associated with the read-out. (2) They allow straightforward calibration as they have a broad smooth responsivity for a wide range of temperatures. (3) The thermal design is simple as there is no heat dissipation in the MMC pixels. These properties have been utilized and demonstrated in many applications by a group in Heidelberg.^10^ The main disadvantage of MMCs as compared with TESs is that they generally require a lower noise amplifier in order to take advantage of the better achievable energy resolution. This means the likely need for one additional stage of amplification of the output signals in the form of a parametric amplifier.^40^

## 4 Read-Out

### 4.1 Basic Circuits and Readout Design

**Fig. 13:**
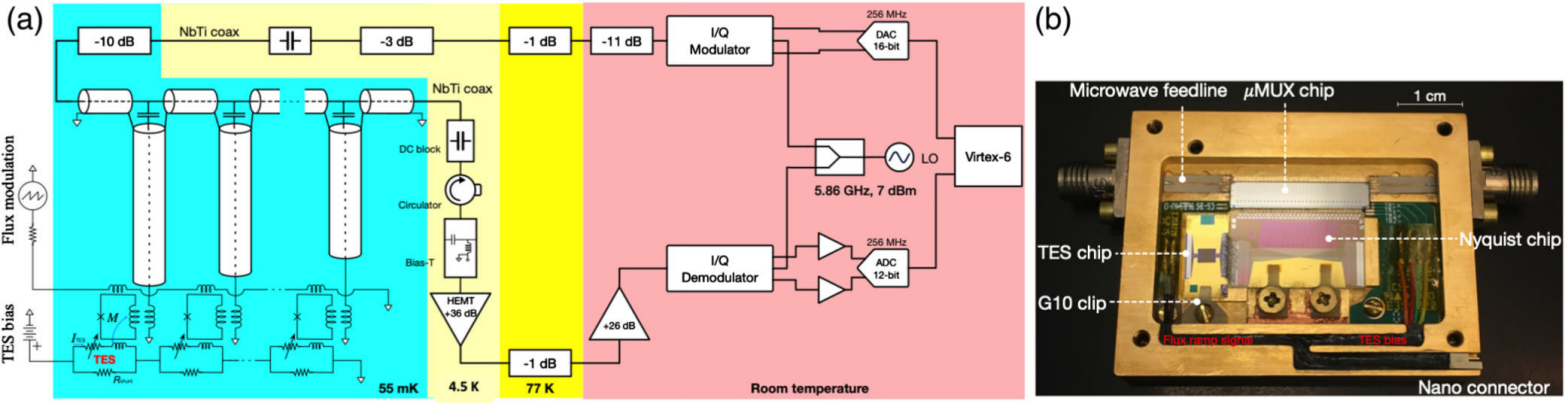
(a) Microwave SQUID multiplexer read-out circuit. (b) Photograph of a prototype set-up for testing arrays pixels with this read-out circuit. The set-up has one microwave read-out channel, capable of reading out 34 separate TESs.

The baseline multiplexed read-out technology for the LXM uses series SQUID microresonator circuits in the GHz frequency range attached to the input feed line to a HEMT, as depicted in Fig. 13(a). Bennett et al.^14^ provide a detailed description of how this read-out technology works, the state-of-the-art, and how they would be developed for the LXM. Here, we very briefly summarize this read-out.

There is a chip at 50 mK on which a large number of resonators are fabricated, with one end of each resonator capacitively coupled to a microwave transmission line and the other inductively coupled to an rf-SQUID. All the resonators have different lengths and therefore different resonance frequencies. Each TES microcalorimeter is voltage biased with a DC current so that any change in TES resistance causes a change in current passing through it. The TES current is inductively coupled to an rf-SQUID. An x-ray photon absorbed in a pixel will cause the current to decrease. This produces a change in the SQUID inductance and consequently a shift in the microwave resonance frequency. The x-ray event thus appears as a phase shift in the SQUID V-Φ. By adding a linear ramp to the flux modulation line, the resonator response to the input signals can be linearized.

The room temperature readout system generates a comb of tones, one for each resonator in the ∼100-MHz frequency range. These tones are mixed with a ∼6-GHz local oscillator signal using an IQ mixer and sent to the microwave SQUID chip to drive the resonators. The transmitted signal is amplified by a low noise HEMT at ∼4 K and again at room temperature, where the signals are then downconverted to the MHz band using an IQ mixer and digitized. An field programmable gate array (FPGA) firmware then channelizes the signals from each resonator and demodulates the flux ramp. Details of the laboratory read-out electronics at room temperature can also be found in Ref. 14. We have studied a flight implementation of the room temperature readout electronics in detail to estimate the necessary processing resources and electrical power needed to run this multiplexer. The results of this study can be found in the accompanying article in this volume in Ref. 41.

The tradeoff for the array design for the number of pixels attached to each microcalorimeter sensor that leads to the currently assumed configuration is not a simple one. There are many different considerations. Previously, the wiring complexity and constraints were a key driver for a decision to strive toward as many pixels per sensor in the Hydra design as possible. However, with the introduction of high-yield, narrow, multilayered wiring, this argument by itself is no longer the most important factor. But by reducing the number of pixels per sensor, we run into another key constraint, which is the surface area needed to read out the greater number of sensors over a given FoV. For each sensor, one needs to consider the surface area per SQUID microresonator in addition to the shunt resistor and Nyquist inductor, in order to determine the total surface of components needed. This is what drives the size of the 50 mK FPA, which in turn determines the size and mass of the cryostat that cools these detectors and their read-out.

For a fixed number of read-out channels, one could consider fewer Hydra absorbers per microcalorimeter sensor (TES or MMC), allowing potentially higher count rate capabilities per pixel and possibly better energy resolution. The bandwidth per Hydra is reduced, and more Hydras can be read out by each channel. However, from the point-of-view of the efficiency of bandwidth usage within the read-out, Hydras generally improve the efficiency of usage at the cost of count rate capability per pixel. A preliminary study has found that to read out a Hydra with 25 pixels attached to each sensor, it requires a slew rate capability that is approximately a factor of 5 larger than for a single pixel, i.e., a factor of 5 less than without the use of the Hydra. Based upon this, our current baseline represents a reasonable compromise between being ambitious in terms of the thermal multiplexing capability of the Hydra and also utilizing the electrical multiplexing of the microwave SQUID resonators to maximum effect.

Similarly, we could consider a greater number of HEMT channels to read out the array, ultimately allowing us to redesign the hydras to have better energy resolution and count rate capability. The down side to this approach is the need then for greater cooling power for the HEMTs at 4.5 K, when the power load from these HEMTs is the main driver for the 4.5-K cooling system. In addition, more room-temperature electronics would be needed, which will then also require more power. The mass of the room temperature electronics would increase as would the mass of radiators needed for this power. This trade-off between the design for the read-out and the cooling system, as well as the power needed for the read-out electronics, all contributed to the design of the proposed architecture. We decided to baseline a maximum of 16 HEMTs to be used to read out the LXM array at one time. We estimate that 10 HEMTs are needed to read out the Main Array and 6 to read out the Enhanced Main Array.^41^ An additional six HEMTs are needed to read out the UHR array,^41^ but this does not need to be operated simultaneously with the Enhanced Main Array.

Table 3 describes our baseline estimate of the multiplexed read-out parameters. Most of the column definitions are self-evident. The energy range for the Main Array here of 6 keV is slightly lower than the required 7 keV in Fig. 2 in the normal operating mode for ease of comparison of models to data at 6 keV. The “margin at max energy” is a comparison of the best estimate for achievable slew-rates for microwave SQUIDs with sufficiently low input-referred current noise to that which is required for reading out the different pixel types listed. Based upon measurements, the best slew-rate estimates for microwave SQUIDs with sufficiently low input-referred current noise is currently 2.3 A/s with spacing with a sample rate of 0.5 Ms/s and bandwidth of 2 MHz. Recent experiments indicate that a resonator spacing that is seven times the bandwidth is desirable to keep nearest neighbor frequency cross-talk negligible. To be conservative, we use a read-out design that maintains 50% margin in this maximum slew rate so as not to require the read-out of each pixel to be right at the limits of capability. In practice, this means that in terms of the read-out, the energy range of the final instrument will likely be much higher than the requirement. In addition, we round up the requirement number of channels/HEMTs to be read out to the nearest even number since we desire to segment the read-out into two completely separate detection chains for reliability. Since the Ultra-Hi-Res Array is operated alone in a separate mode from the main and Enhanced Main Array, a maximum of 16 HEMTs will operate simultaneously. The number of coaxial cables needed for this read-out configuration is 44.

### 4.2 High-Electron Mobility Transistors

HEMT amplifiers exist with input noise temperatures as low as 1.8 K over the 4 to 8 GHz band. Several commercial vendors supply HEMT amplifiers. Various HEMT models cover lower and higher frequencies and narrower and broader bands with different noise temperatures. Commercial low noise amplifiers (LNAs) exist that can provide gain after the HEMT stage. We continue to refer to the 4.5-K amplifier as the HEMT architecture even though, strictly speaking, there are several amplifiers. The architecture includes the number of stages, the thermal locations of the stages, as well as the gain, noise, power dissipation, and linearity of each stage. Linearity plays an important role in setting cross-talk levels. Some of these properties are in tension. The connections between stages are important, and different types of connections are possible and suitable at different temperatures.

In our baseline configuration, we have assumed the properties of a two-stage HEMT operating at 4.5 K that is available commercially and which we believe is sufficient to boost the signal to the required levels before reaching the room temperature electronics. The HEMT available from low noise factory, “LNF-LNC4_8DRPA s/n 009Z 4–8 GHz Cryogenic LNA,” provides a noise temperature of ∼3 K when operated with 1 mW of power. It provides a gain between 34 and 38 dB with 1 mW of power.^42^ Development will be needed to determine whether this HEMT alone will be sufficient, or whether a further stage of amplification at the next thermal stage is needed, for instance, the 15 K stage of the LXM cryostat.

The HEMTs are considered an important technology, where investment will be needed to provide us with flight-qualified versions with suitable performance and heat load. Although commercial HEMT amplifiers exist with excellent properties, Lynx may benefit from, or require, HEMTs with different design optimizations. The power dissipation of individual HEMTs is of order 1 mW and a 16 HEMTs will be on at a time, so the HEMTs will have a significant effect on the thermal budget of the LXM. While HEMTs are not seen as an item that needs immediate attention, at some point in the development cycle, likely in the pre-Phase-A period, development work will be required. There could be some benefit if a company with state-of-the-art HEMT development capabilities could develop a flight HEMT with the properties desired by the LXM as part of a Small Business Innovation Research program.

### 4.3 Current Technical Readiness Level of the Readout

The current TRL with respect to Lynx requirements, as assessed against the read-out definitions described in Sec. 9, is TRL-3. The operational principle of microwave SQUID read-out has successfully been demonstrated and agrees within expectation well within a factor of 2, as described by Ref. 14. They have been shown to operate when reading out gamma-ray transition-edge microcalorimeters,^13^ and when reading out x-rays in the 0.2- to 7-keV band.^43^ To reach TRL-4, we will need to demonstrate the microwave SQUID readout when reading out the different LXM pixel types and closely agree with the expected performance. The NIST Quantum Devices group in Boulder, collaborating with the x-ray microcalorimeter group at NASA, have recently been awarded a new APRA award specifically to carry out this development. Once the detector arrays shown in Sec. 3 have been tested, they will be provided to NIST for them to carry out microwave read-out tests on to establish TRL-4. This group will continue to optimize the design and performance and participate in the large demonstration needed to establish TRL-5 by 2024, as described in Sec. 7.3. The Irwin Laboratory at Stanford will also continue to develop microwave read-out technology, investigating a number of innovative ways to ease and improve the microwave readout for the LXM using techniques called spread spectrum sharing and frequency tone tracking.^14,41^

## 5 Cooling System

### 5.1 Cooling Concept

**Fig. 14:**
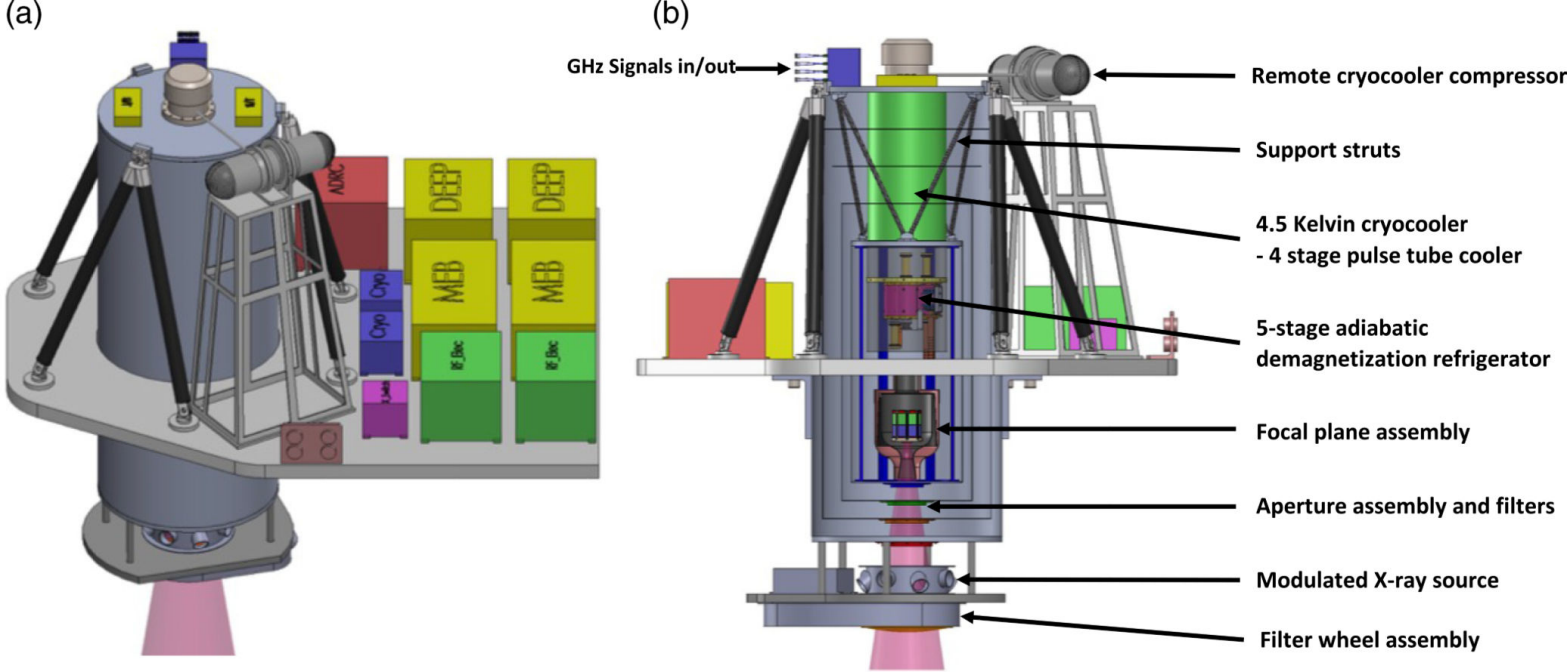
(a) Overview of the LXM cryostat and read-out electronics. The compressor is mounted independent of the cryostat and connected to the cryostat through a flexible transfer tube. All of the electronics boxes shown in the LXM block diagram (Fig. 18) are visible. (b) Side-on view of the LXM, including a cross-sectional view of the cryostat. The x-ray enter the cryostat from the bottom. The filter wheel and MXS (with its electronics) are located a small distance below the bottom of the main cryostat on a separate mounting plate, which is attached to the main cryostat. The height of the main cryostat shown is 1.43 m and the diameter is 60 cm.

The LXM will be cooled to 50 mK using a cryostat that takes advantage of the heritage of the mature Hitomi ADR technologies. A detailed description of the cooling options under consideration for the LXM is given in Ref. 19. The preliminary design for the cooling system is shown in Fig. 14.

The key components in the cooling system are the cryocooler, which cools the detectors from room temperature (assumed to be 283 K in our thermal design) down to 4.5 K, and a multistage ADR that provides continuous cooling down to ∼50 mK. A key feature of the design is that the cryocooler compressor, rotating valve, and all moving parts are supported on a separate stand from the cryostat to minimize vibrational coupling. It is only coupled to the cryostat through a flexible tube, allowing for the cryostat to remain vibrationally isolated. Details of the LXM read-out electronics are also shown in Fig. 14. The dominant heat loads from the detection chain at 4.5 K come from the HEMTs, which are estimated to dissipate 16 mW, and the harnesses, which are estimated to conduct 3 mW. The ADR is estimated to generate 4 mW, and the cryostat support struts and radiation are expected to produce heat loads of 2.5 and 0.1 mW, respectively. The total heat load is then ∼25 mW. Following the suggested margin by NASA for a program at this stage of development of 100% for a cooling system, we have designed our 4.5 K cooling system based on a heat load of 50 mW.

The preliminary cryostat design is based on a desire to minimize the diameter of the cryostat around the detectors, in order to allow the detectors for the x-ray grating spectrometer to be located as close as possible to the central axis of the x-ray beam, allowing for simultaneous observations. The diameter of the cryostat in this preliminary design is 60 cm. At the bottom of the cryostat, there is a gate valve and an aperture assembly to enable x-rays to directly pass through the microcalorimeters while incorporating thin-film filters to block infrared and optical photons. A detailed description of the aperture assembly and the filter is described in Ref. 44. Outside of the gate valve, the LXM also includes an external filter wheel and a MXS that is capable of providing pulsed x-ray lines at multiple energies and is similar to that used on Athena’s X-IFU^5^ and Hitomi’s SXS^15^ for in-flight calibration. These are also described in Ref. 44.

### 5.2 Cryocoolers for the LXM

There are a number of different options for providing 4.5 K cooling,^19^ but for now, we have based our preliminary design based on the use of ACTDP 4-stage (Mega4–1) pulse tube cryocooler, of the type that is developed by Lockheed Martin.^45,46^ This cryocooler is currently at TRL-4, although many components of the cryocooler are at a much higher TRL.^19^

We have also been considering a Turbo-Brayton cryocooler for the 4.5 K cooling of the type that is developed by Creare.^47^ The advantages of this type of cryocooler is the inherent lack of vibration generated by the cryocooler, as it is based upon the use of extremely low-mass moving parts moving at speeds in excess of 1 kHz, and the high mechanical reliability from the use of gas bearings and clearance seals that prevent mechanical contact and thus eliminate wear. Its operation is completely independent of gravity and estimates for the cooling power at 4.5 K are in the range of 200 mW.

This type of cooler is not currently baselined because it has not yet reached TRL-4. Its development is currently based upon an existing 10 K cooler currently at TRL-5, and a 4 K cooler under development for the Navy. A version with a third stage of cooling to reach 4.5 K as proposed for the LXM is still in development. Therefore, although many components of this cooler are at TRL-5 and TRL-6, the lowest temperature turbo alternator is currently at TRL-3.

### 5.3 Multistage Adiabatic Demagnetization Refrigerator

**Fig. 15:**
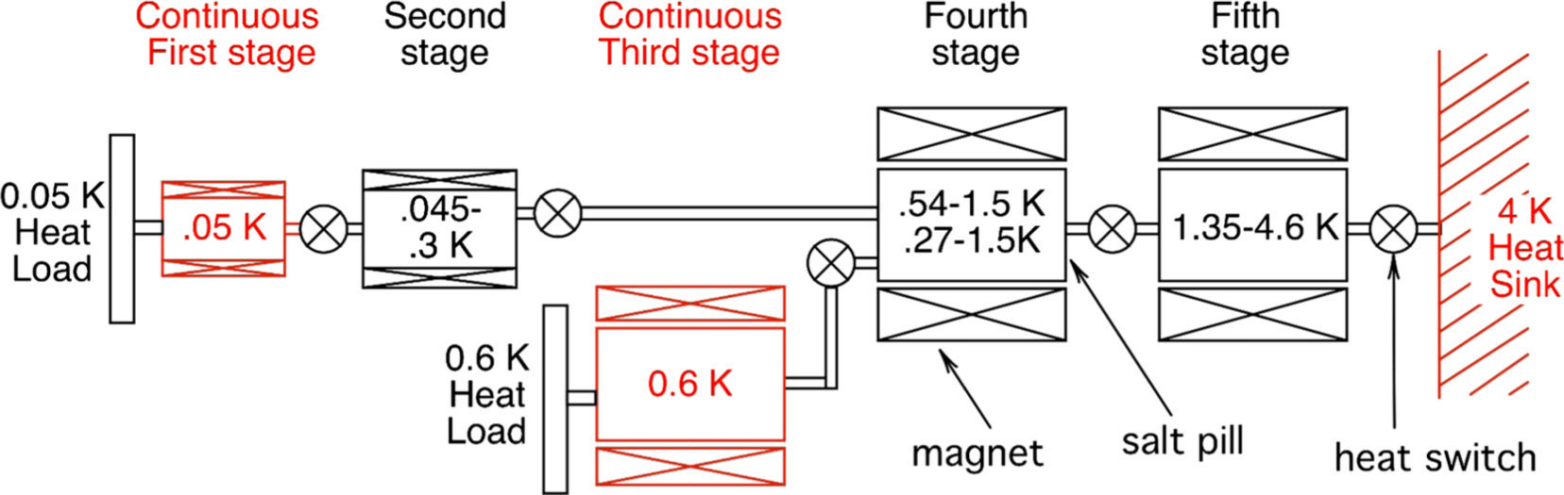
The LXM multistage ADR design that produces the required cooling power at 50 mK and 0.6 K continuously, assuming a 4.5-K heat sink temperature. It consists of five different salt pills and five heat switches. Its salt pill is surrounded by its own magnet.

The LXM ADR provides continuous cooling at 0.6 K and 50 mK using a five-stage ADR, as shown in Fig. 15. The design of this ADR and its controller are based on the three-stage ADR used for Hitomi. It is currently in development at GSFC^18^ and is expected to reach TRL-5 by the end of 2019. The heat loads from the detection chain and thermometry dissipation and harness conduction and from the suspension system are estimated to 124 *μ*W at 0.6 K and 3.0 *μ*W at 50 mK. The ADR is designed to meet these cooling requirements continuously with 100% margin, in accordance with NASA guidelines for cryogenic cooling margins. More details on the design of this ADR are described in Ref. 19. A preliminary drawing showing how it is incorporated into the LXM is shown in Fig. 14. The average amount of power produced by the ADR over its cycles of continuous cooling is 4 mW. The power produced varies during the cycle, but the ADR is thermally engineered to create a uniform heat load on the cryocooler.

## 6 Focal Plane Assembly

### 6.1 Focal Plane Assembly Design Concept

**Fig. 16:**
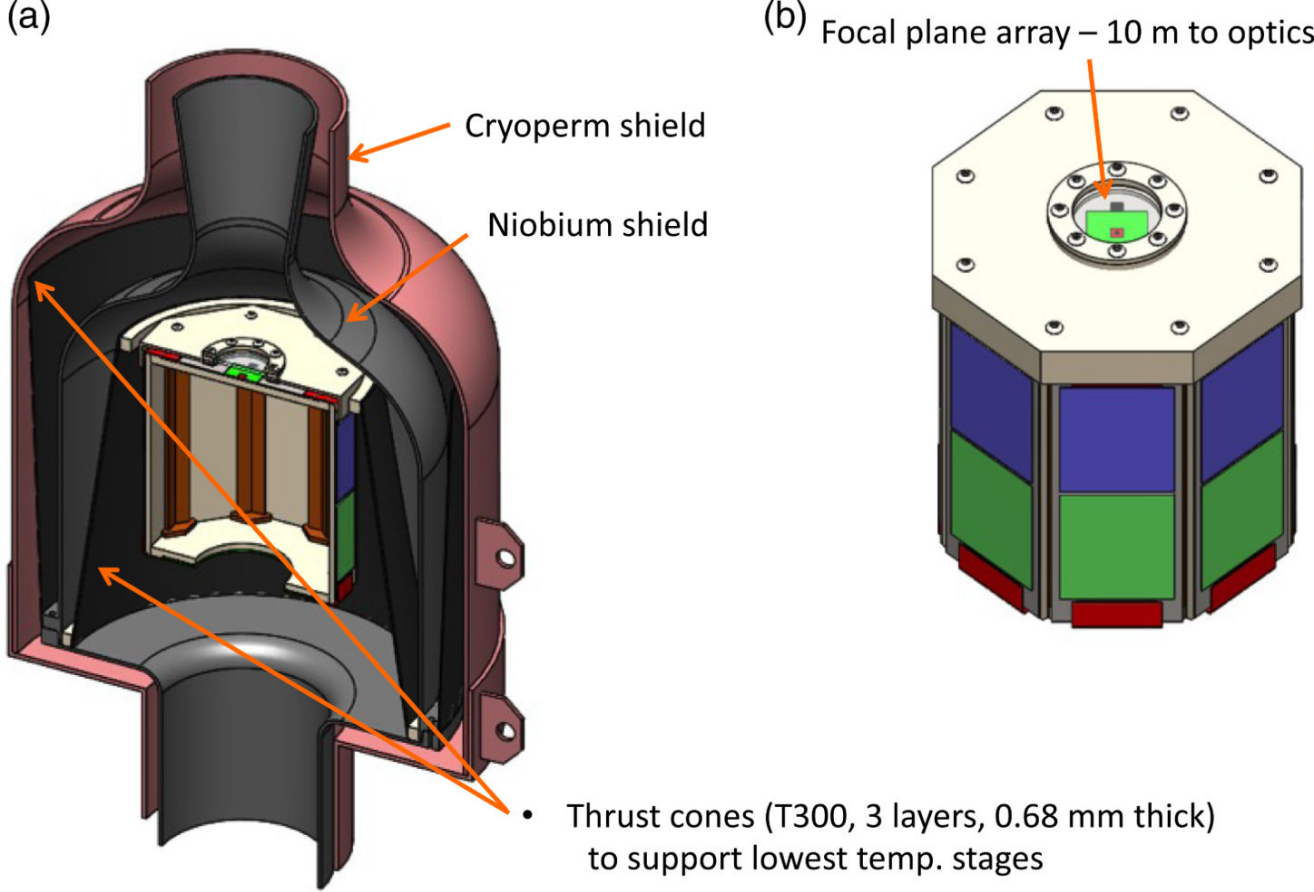
Preliminary LXM FPA design. (a) Cross-section of the FPA. The high-magnetic-permeability cryoperm shield is at 4.5 K and the superconducting niobium shield is at 0.6 K. (b) Angled view of the FPA 50 mK stage. The main, enhanced main, and UHR arrays are visible on the top surface through am infrared blocking filter, which is almost transparent in this figure. The read-out components are on each of the eight side panels.

The preliminary FPA design for the LXM is shown in Fig. 16. Because the LXM FPA is similar in size to the Athena X-IFU FPA, it assumes leveraging much of the same technology in the mechanical design, thermal design, magnetic shielding, and design of the anticoincidence detector. These are described in Refs. 20, 48, and 49. Here, we emphasize some of the differences.

One of the key differences compared with the Athena X-IFU is the higher heat load estimated for the LXM at 50 mK, and also, therefore, the associated higher cooling capacity ADR. This difference allows us to consider a mechanical support for the 50 mK stage that uses fiberglass thrust tubes (slightly canonical) to support the 50 mK stage from the 0.6 K stage, and the 0.6 K stage from the 4.5 K stage, instead of following the more traditional approach for lower cooling power ADRs using low thermal conductivity Kevlar strings. These are shown in Fig. 16(a). We have assumed the use of three layers of 0.68-mm thick T300 (arranged in 60/0/60 orientations) and use of a tube 17 cm in diameter and 19 cm long.

Mechanical models have investigated whether such a tube can withstand the mechanical vibrations of launch, assuming 20 g’s along each axis and designing the structure to ensure that the first mode of vibration is greater than 50 Hz. In this analysis, the mass of the 50 mK stage was assumed to be 7 kg, which is many times higher than the mass of this stage in our current design. The first modes of vibrations were verified to be greater than 50 Hz. We modeled the sensitivity to buckling in both the lateral and axial axes with 20 g’s and found that no buckling is expected. We have included a calculation of the heat loads associated with these thrust tubes between each of the stages, and these contributions have already been included in the thermal budget described in Sec. 5.3. Other geometries for these stages will need to be investigated, but we have concluded that this basic type of geometry will work.

It is important to confirm that the area of chip components for the read-out on the side panels is sufficient for reading out the entire array. Currently, the microwave SQUID resonator cell sizes are close to 1 mm^2^. It is fairly straightforward to see how this could be shrunk to something near 0.5 mm^2^. The shunt resistors and Nyquist inductors in the TES bias circuit also take up an appreciable area. In current designs, the Nyquist inductor is around 0.7 mm^2^ area and a 0.2 mΩ shunt is 0.3 mm^2^. Thus, we have conservatively assumed a footprint to read out for each TES consisting of 1 mm^2^ for the SQUID resonator and 1 mm^2^ for the shunt and Nyquist inductor. These two types of component areas are represented by the blue (Nyquist inductor and shunt, ∼1000 per panel) and green (microwave resonator, ∼1000 per panel) regions of the side panels in Fig. 16(b).

In order to read out all of the signal chains, there needs to be a careful consideration of how to route all of the signals through coax cables, microstrip flex, and regular twisted pairs of wires, and the levels of associated heat loads, how to make contact between the difference sorts of harnessing, and how heat-sinking is achieved. Here, we concentrate on the practicality of the nonstandard harnessing, which is mainly for the signal lines with GHz frequency signals. For the signals between the HEMTs and room temperature electronics, fairly standard semirigid coax cables are baselined, just are used in numerous laboratories worldwide. From the HEMTs at 4.5 K to the detectors at 50 mK, the options for the cabling are miniature superconducting coax cables, also in widespread use now, and cryogenic microstrip on Kapton microwave flex, as has been demonstrated in a few laboratories, such as reported in Ref. 50. We have estimated the heat loads at 600 and 50 mK based on the use of 0.085” diameter NbTi coaxial cables, even though for fragile smaller diameter, NbTi coaxial cables are available (currently down to 0.035” diameter). Superconducting versions of such microwave flex should be feasible. Although requirements can be met using coaxial cables, if the flex is developed, it will increase the margin relative to the thermal requirements. Standard semirigid coaxial cables are baselined to be used between the HEMTs and room temperature.

### 6.2 Contacts and Harnessing

**Fig. 17:**
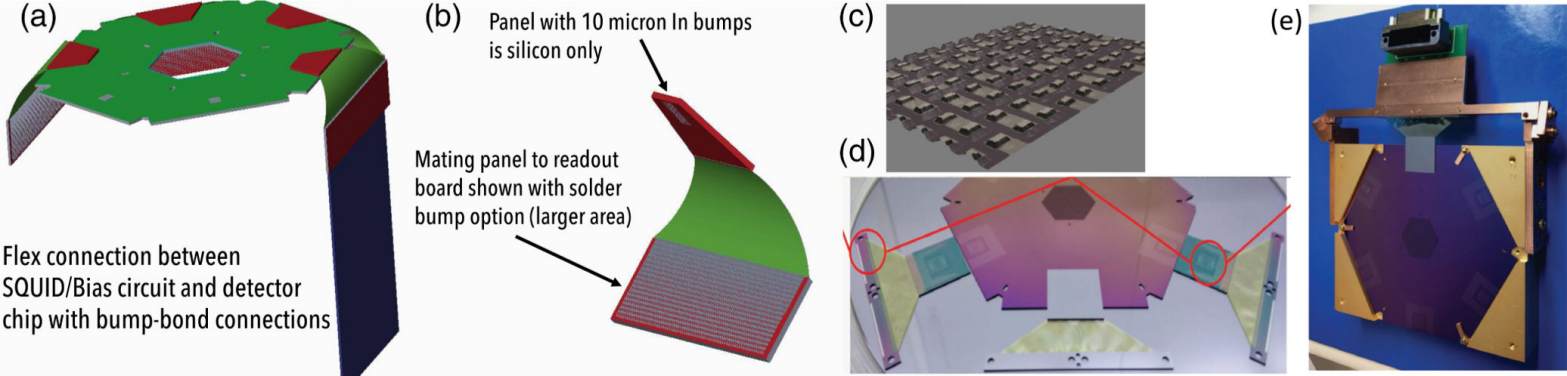
(a) Illustration showing how bump-bonded connections are made between the detector chip and the side panels containing the readout. (b) Illustration showing the geometry of the flex and bumps. (c) Scanning electron microscope image of some prototype indium bumps fabricated and bump-bonded at GSFC. (d) Photograph a hexagonal detector chip connected to several silicon chips with wire-bond pads using superconducting microstrip flex and bump-bond connections. (e) Set-up for testing the bump-bonded connections between two chips through flex with superconducting microstrip.

One of the key issues in the design of the FPA is how to make connections between many TES channels on the detector chip and their low-temperature microwave SQUID read-out. It is highly desirable to route the signals around the corner in the geometry of FPA shown in Fig. 16 due to considerations of the mass of the FPA and its magnetic shielding. These signals are only at frequencies ranging from DC to tens of kilohertz, and therefore, do not require GHz flex. However, they do require something like flex with microstrip geometry to maintain good immunity to electromagnetic induction pick-up and to maintain a small footprint. To make contact at each end of the flex, bump bonding is the most reliable and well-established technology to use for very large arrays. Several groups have developed such microstrip flex for the read-out of TESs. In Fig. 17, we show how both the flex and the bump-bonding has been developed at GSFC.^51^ The images in (a) and (b) describe the geometry being used for the flex and bump-bonding, image (c) is a scanning electron microscope image of some test indium bumps, image (d) is a photograph of a detector wafer and a chip with the a silicon chip with fanned-out wire-bond pads and a superconducting microstrip flex in-between, and image (e) shows a fixture we are using to make controlled bends of the flex and to make resistance and critical current tests on the microstrip flex through bump-bond connections.

## 7 System Design

### 7.1 Block Diagram

**Fig. 18:**
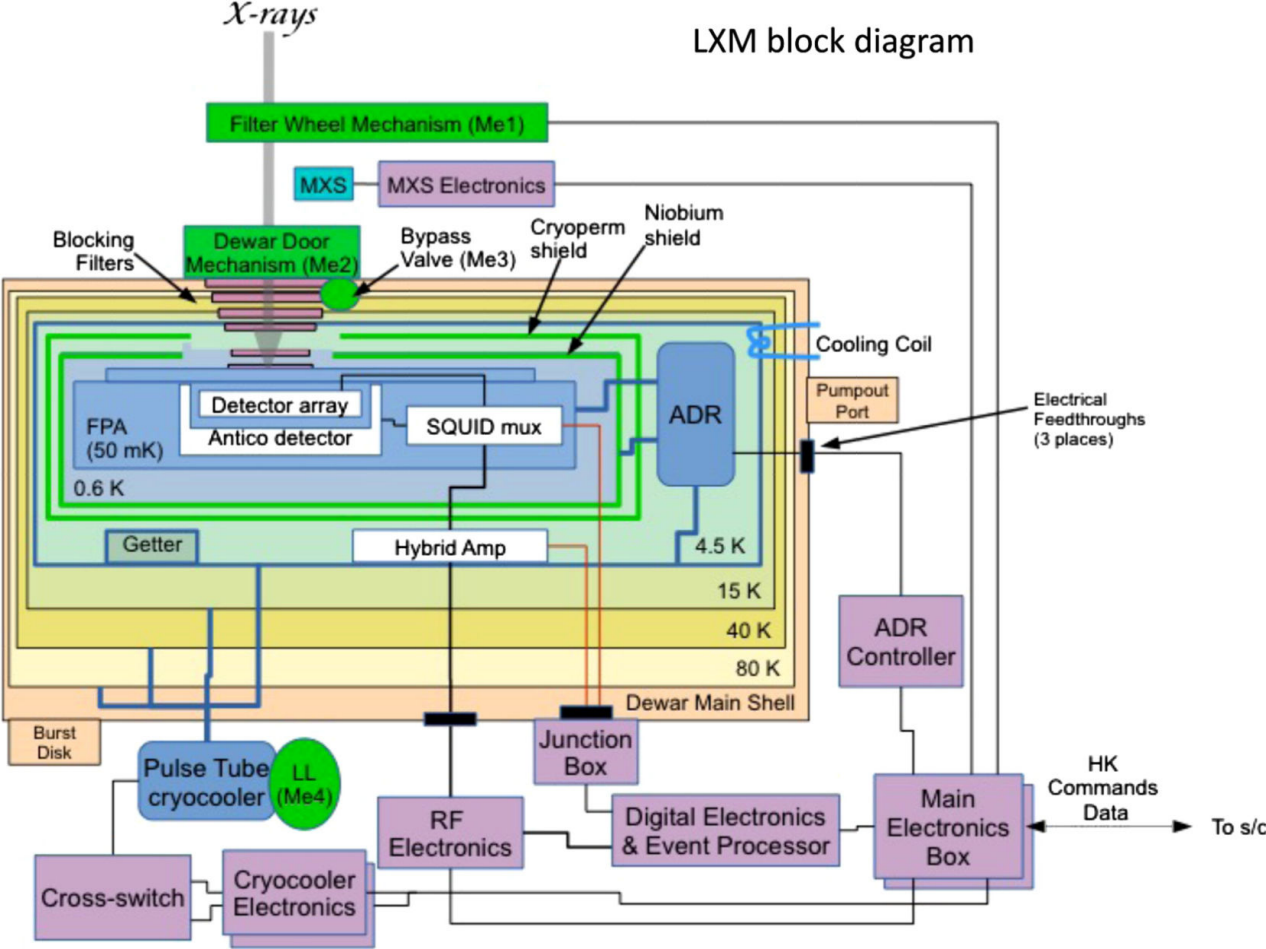
Block diagram of the LXM. The cryocooler and MEB consist of two redundant boxes. The DEEP box is actually two separate segmented boxes.

The block diagram for the complete LXM is shown in Fig. 18. This diagram shows all of the components of the LXM that have been described in this paper and also in the accompanying papers in this volume related to the LXM. It shows the two redundant electronics boxes that are assumed to be needed to read out the cryocooler, and the redundant main electronics boxes (MEBs). These MEBs have a microprocessor and several conditioned power sources and control signals for all of the various electronics boxes and mechanisms. It is also responsible for sending out the data signals and receiving commands from the space craft. Redundancy is needed to maintain high reliability of the instrument. The digital electronics and event processor (DEEP) also consists of two boxes, providing segmentation of the read-out.

### 7.2 Mass/Power/Data Rate

We have developed a detailed master equipment list for the LXM working together with the Goddard Space Flight Center Instrument Design Laboratory allowing us to make estimates for the required mass, power, and data rate. Our current best estimate for the mass is 468 kg. This is dominated by the mass of the cryostat (164 kg) and the mass of the electronics boxes (146 kg). Other significant contributions to the mass of the LXM comes from the thermal system (72 kg), which is mainly the heat pipes that connect the cryocooler and electronics boxes to the radiators. The mass of radiators is considered as part of the Lynx spacecraft. The harnesses are estimated to be 34 kg, and then, there are a number of other smaller contributions that we estimate to total up to 52 kg.

The total power needed for various components of the LXM is summarized in Table 4. The total average power needed during normal operation is ∼1.6 kW. The total power is largely driven by the power needed for the cryocooler and the DEEP electronics box. We have been conservative about our choice of FPGAs, and it is quite likely that future processors will become available that could reduce the power needed. Similarly, it is very possible that future cryocoolers will require less power. As we reduce the power required for the LXM, we can reduce the size and mass of the radiators surrounding the LXM around the outside of the Lynx Integrated Science Instrument Module, already sized to have a margin of 40%. The LXM is assumed to launch warm and only needs about 10 W of housekeeping power until the observatory is en route to L2.

The assumed maximum data rate for the LXM is based on a total maximum count rate for the whole array 100,000 cps and the assumption that 80 bits are needed to describe each event. Therefore, the LXM has a maximum data rate of 8 Mbps. When looking at the brightest sources, such as Sco X-1, and utilizing the defocused array option, the instantaneous data rate may be higher. In such cases, we assume that (a) other instruments are turned off and (b) there is a limit on exposure such that the total volume of data collected is <240 Gb/day, which is the telemetry limit of Lynx per day.

### 7.3 Development Plan

With over 30 times more pixels than the X-IFU, the LXM concept appears very demanding; however, as described in Secs. 3 and 4, there has been significant technology investment and development, which has led to a number of recent breakthroughs that makes the concept feasible. To summarize, three of the major breakthroughs are as follows:

- The successful demonstration of a type of microcalorimeter in which many pixels are attached to a single temperature sensor called hydras. The LXM consists of hydras with as many as 25 pixels attached to a single sensor.
- The integration of microcalorimeter arrays with fine-pitch, multilayer, superconducting wiring buried beneath a planarized substrate.
- The development of SQUIDs coupled to microwave resonators has dramatically advanced the number of sensors read out on each signal chain, from the range of tens (for the X-IFU) to hundreds. This has leveraged readout technology developed for infrared detectors and, in particular, the approach pioneered for the development of microwave kinetic inductance detectors.

Recent advances have been so successful that fully wired microcalorimeter arrays, with over 55,000 microcalorimeter pixels (spread over the three different pixel types needed for LXM), have been fabricated. Results from large prototype arrays using TESs have shown that the performance requirements for all pixel types are feasible. And a full-size LXM array is under development, with fabrication completion scheduled for late 2019.

**Fig. 19:**
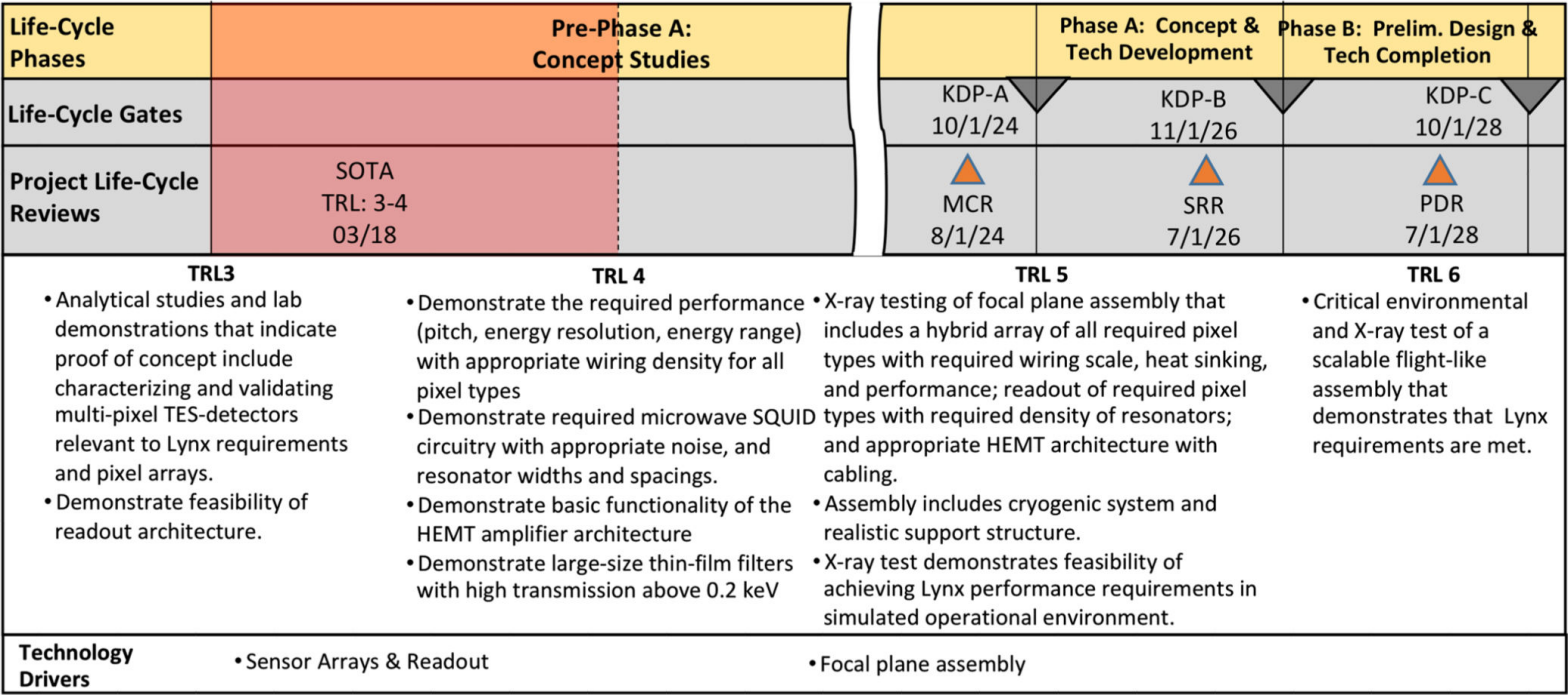
The schedule for the LXM development and a very brief summary of the plan for demonstrating the TRL advancements.

For each of the important technologies that need to be developed for the LXM, we have summarized our plan to evolve these technologies to TRL-5 in time to begin Phase-A in 2024. Because the path of this development is by now well-known within the low-temperature detector community, and based on the above already achieved breakthroughs, the degree of difficulty for the subsequent advancement of this technology is greatly reduced compared with many other less mature technological approaches. We are confident that we have a solid plan for the required TRL advancement. This plan is summarized in Fig. 19. All the critical technologies needed for LXM will achieve TRL-4 by the start of pre-Phase-A (currently assumed to be approximately 10/1/21), funded through ongoing existing research and development programs. All the critical technologies will achieve TRL-5 prior to the end of pre-Phase-A (currently assumed to September 2024), with 9 months of margin. And the critical detector and readout technologies will achieve TRL-6 by the end of Phase-A (currently assumed to be September 2026) through a TRL-6 demonstration unit, with 7 months of margin.

While some of the technologies are currently assessed as TRL-3, the existing ongoing programs are expected to achieve TRL-4 in all of these by September 2021. The LXM detector development assumes a continuation, assuming a continuation of the two possible detector options through the first 3 years of pre-Phase-A development, after which a decision will be made to continue with the current baseline (TESs) only or switch to MMCs. Both the TES and MMC development are currently supported by NASA. No major infrastructure upgrades are needed, as the development will leverage the substantial investment that has been made for the development of detectors for the X-IFU. NASA also supports a program developing buried wiring for both TES and MMC detector development. One of these two detector technology options will be chosen at the end of pre-Phase-A.

NASA also already supports a number of groups developing microwave SQUID readout technology. There will be a need for the development of low-noise high-dynamic range HEMTs that are space-qualified and packaged suitably for this application. There will not be too much FPA development in pre-Phase-A, just preliminary design development since the plan will be to highly leverage the technologies currently in development for the Athena X-IFU. Once in Phase-A, the development will need to ramp up substantially to evolve a specific design and plan for the engineering and flight models of the LXM FPA. It is not expected that bump-bonding will need a separate research effort in pre-Phase-A since the TES and MMC detectors’ needs will be met within the detector development programs. For TESs, it will highly leverage the bump-bonding and flex development that has already taken place and continues for the backup readout option for the X-IFU.

While the FPA and harnessing represent a critical and complicated engineering focus area, we do not consider this technology to be critical enough to track its TRL. Based up existing technology developments for the Athena X-IFU, and other technologies that are likely to continue to evolve for ground-based applications for a wide variety of applications, we believe that the LXM FPA is feasible and will require greater engineering resources at a later stage of the project development, once the project is in Phase-A. Some significant blocking filter development is expected throughout pre-Phase-A as the optimization of the filter design will likely produce a high return on investment in terms of evolving the TRL of infrared photon blocking filters with the highest throughput for low energy x-rays. Some filter design concepts will need to be demonstrated. Once in Phase-A, the filters budget will need to ramp up in order to build and demonstrate a set of filters compatible with the FPA and cryostat design.

For the cryostat development, the roadmap includes the development of a design that meets the thermal and structural requirements of LXM that would be completed during Phase-A. The cryocoolers will require significant resources to advance their TRL to TRL-5. For the two cryocooler options identified, this is expected to be relatively straightforward, but the cost is relatively high. This will be an important investment that should begin in pre-Phase-A. The plan for the TRL advancement for the cryocoolers is to advance both cryocooler options to TRL-5 during pre-Phase-A development in the early 2020s, with a decision on the baseline being made at the start of Phase-A. For the chosen technology, first, a bread-board model will be developed to establish TRL-6, before building an engineering model, and then a protoflight and flight spare cryocooler. The cost of this development plan and all of these models have been included in our grassroots estimate. It will be important to support the evolution of the continuous ADR that already has an existing program to take this technology to TRL-5. We plan to adapt and optimize this technology specifically to meet the LXM requirements.

The future model philosophy of the LXM is based on the approach that was followed by the SXS instrument on Astro-H, planned for the resolve instrument on XRISM, and is similar to the approach planned for the Athena X-IFU. It is based on the development of an engineering model (EM) and a proto-flight model (FM), with selected subsystem flight spares but no complete instrument spare. There is no qualification model at the instrument or subsystem level (as is planned for the X-IFU), but it is planned that the EM will undergo extensive qualification testing beyond the typical level of an engineering development unit in order to space-qualify the design.

It is critical to the success of this plan that the schedule for the detectors and readout components allows work on these key components relatively early in the program, with work on the arrays and read-out for the EM and FM prior to the overall mission preliminary design review and critical design review, respectively, following key-decision-point reviews for just these two items. This is due to the long lead times associated with the design, fabrication, and testing of these components, which must all be complete prior to integration and testing within the FPA. The FPA, in turn, needs to be integrated and tested with the rest of the instrument. The long sequence typically consists of approximately a year of development to produce the detector, and another year to integrate and test within the FPA. In order to address the lessons learned from the EM development, a critical element of the plan ensures that the FM does not begin until the EM FPA has been tested. For this to happen, in order to align it with the development schedule for the rest of LXM and the rest of the mission, the detectors and readout should be at TRL-6 by the end of Phase-A (9/30/26). Our development plan achieves this with 9 months of margin.

## 8 Conclusion

We have developed a detailed design concept for the LXM that meets the scientific requirements for several of Lynx’s key science measurements. There are two promising detector technologies with the potential to meet all of these requirements. We baseline the TES technology based on its greater technical maturity and demonstrated performance. MMCs are a very capable parallel detector technology being developed as well. The current TRL of the detectors is 3, but measurements and development plans exist that could allow TRL-4 to be demonstrated within 2019. We have described a read-out technology that is rapidly increasing in maturity and appears to be very capable of meeting the demands of the baseline detector. We have a good understanding of the FPGA and power resources needed to read out the detectors, which are not excessively large when compared with the total resources needed on this proposed flagship mission. The cooling system baselined is reasonably mature, and several parallel options for the cryocooler are available. We have baselined a relatively simple and low-cost cryocooler that is at TRL-4, although there are a number of somewhat more complex higher TRL cooling systems available should the four-stage pulse-tube cooler option not advance to TRL-5 and 6 as expected. LXM’s required spacecraft resources are well within the available resources of the Lynx spacecraft. We have described a solid technology maturation plan to be ready for Phase-A in the mid-2020s, and thus, well-phased with the foreseen schedule for the Lynx mission concept.

## Figures and Tables

**Table 1:** This table summarizes pixels in each of the three subarrays.

Arrays *T*_c_ = 65 mK	FoV	Type	Hydra factor	Pitch	Pitch (*μ*m)	Thickness (*μ*m)	ΔE FWHM	# of pixels	# of sensors	Max slew rate for max *E* (A/s)
Main Array	5′	5×5 hydras	25	1″	50	4	3.0 eV	86,400	3456	1.5
Enhanced Main Array	1′	5×5 hydras	25	0.5″	25	4	2.0 eV	14,400	576	6
Ultra-Hi-Res Array	1′	Single pixels	1	1″	50	1	0.3 eV	3600	3600	0.85
Total								104,400	7632	

**Table 2:** The main physical parameters for each of the three-pixel types based on models of these pixels.

Arrays *T*_c_ = 65 mK	FoV	Type	*C* (pJ/K)	*G* (pW/K)	Tau (ms)	Count-rate (cps)	TES power (pW)	Shunt power (pW)
Main Array	5′	5×5 hydras	0.82	155	2.5	10	2.1	21
Enhanced Main Array	1′	5×5 hydras	0.20	65	0.674	40	0.52	5.2
Ultra-Hi-Res Array	1′	Single pixels	0.013	22	0.324	80	0.173	1.73

**Table 3:** This table describes the number of resonators on each microwave channel and the calculated performance parameters needed of the microwave read-out for the three different pixel types.

Arrays	Input referred noise $\left(\mathrm{pA}/\sqrt{\mathrm{Hz}}\right)$	# of TESs	Max slew rate for max *E* (A/s)	Energy range (keV)	Sample rate (MS/s)	Res. Width (MHz)	Res. Spacing (MHz)	# of res. per HEMT	uMUX power (nW)	Margin at max energy	HEMTs needed	Total HEMTs
Main Array	27	3456	1.5	6	0.5	1.4	10	400	4	1.5	8.6	10
Enhanced Main Array	27	576	6	6	2	5.6	40	100	1	1.5	5.8	6
Ultra-hi-res array	27	3600	0.85	1	0.3	0.86	6	667	7	1.6	5.4	6
Total		7632										**22**

**Table 4:** Table showing the power needed for each of the different electronics boxes in the LXM.

Power source	Power (W)
DEEP boxes	615
Cryocooler	653
MEB	20
RF electronics boxes	141
Junction box	5
MXS control electronics	20
ADRC electronics box	60
Operational heaters	50
Total	1564
Total with 40% margin	2190

## 9 Appendix: Detailed Requirements

Table 5 describes the detailed requirements, science drivers, and comments on the requirements for each of the three sub-array types, the Main Array, the Enhanced Main Array and the Ultra-Hi-Res Array. Table 6 list technology development milestones for the LXM generated by the LXM working group for TRL-3 through TRL-6. These are consistent with the NASA TRL definitions considering the requirements of this instrument.

**Table 5:** This table lists the requirements for each of the three sub-array types, the Main Array, the Enhanced Main Array, and the Ultra-Hi-Res Array.

Main Array (excluding central arc min)			
LXM Main Array parameter	Requirement	Science driver	Notes

Energy range (keV) Minimum Maximum	0.2 7 keV for 3 eV normal mode ~15 keV for 5 eV hi-E mode	Need to extend energy range to determine continuum for studying various AGN. At low end to see low temp. thermal emission or low energy nonthermal sources.	Low-res mode achieved by increasing the bath temperature
Quantum efficiency	Area fill factor >90% Vertical QE >80% at 6 keV	Maximization of effective area (counts)/minimization of observation times.	Limited by: - area fill-factor, - IR blocking filter design - absorber thickness 4 *μ*m Au absorbers baselined. - If 2 eV possible at low energies with 2 *μ*m Au, that is ok.
FoV	5 × 5 arc min (1.5 cm × 1.5 cm)	Characteristic size of many extended objects (SNR, galaxies and clusters of galaxies) for high-res imagining and spectroscopy	A few larger images can be acquired through mosaicking observations
Pixel size (arc sec)	1 × 1 (50 *μ*m × 50 *μ*m)	Removal of point sources to minimize background in diffuse emission, to study arc second scale features such as shocks and filaments, and point sources in crowded regions (XRBs and stars)	Smaller pixels off-axis not essential and would require too many sensors to read out
Energy resolution	• 3 eV (FWHM) (hi-res) • 2 eV (FWHM) (Goal - hi-res) • 5 eV (FWHM) (mid-res) • 10 eV (FWHM) (low-res)	Line-separation/velocity accuracy to determine energetics and dynamics of plasmas	Sufficient for required plasma diagnostics and energetics
Count-rate capability	• 10 cps/hydra (0.1 mC) hi-res mode (per 25 contiguous pixels) • 40 cps/hydra (0.4 mC) mid-res • 150 cps/hydra (1.5 mC) low-res	Accommodation of typical flux of interesting sources	Essentially the count-rate per point source
Timing resolution Timing accuracy	2 *μ*s 50 *μ*s		
Enhanced Main Array (central arc minute, excluding high-res inner array)			
LXM Main Array parameter	Requirement	Science Driver	Notes

Energy range (keV) Minimum Maximum	0.2 7 keV for 3 eV normal mode ~15 keV for 5 eV hi-E mode	Need to extend energy range to determine continuum for studying various AGN. At low end to see low temp. thermal emission or low energy nonthermal sources.	Low-res mode achieved by increasing the bath temperature
Quantum efficiency (keV)	Area fill factor >80% Vertical QE >95% at 7 keV	Maximization of counts/minimization of observation times	Limited by: - area fill-factor, - IR blocking filter design - absorber thickness (6 keV requirement would be good.)
FoV	1 × 1 arc min (3 mm × 3 mm)	Minimum size of fine structure in objects requiring extremely high angular resolution, such as jets, centers of galaxies, and cores of clusters of galaxies	
Pixel size (arc sec)	0.5 × 0.5 (25 *μ*m × 25*μ*m)	Study of sub-arc second scale features such as shocks and filaments, and point sources in crowded regions (XRBs and stars). Study of distribution of AGN within and around groups/clusters, removing AGN, the study of thermodynamic properties of cluster gas. Feedback in groups and clusters	
Energy resolution	• 2 eV (FWHM) (hi-res) • 4 eV (FWHM) (mid-res) • 10 eV (FWHM) (low-res)	Line-separation/velocity accuracy to determine energetics and dynamics of plasmas. For *z* = 1, when SMBH growth and AG feedback at peak, features red-shifted from 3 to 4 keV, requiring 1.5 to 2 eV resolution	1.5 eV possible
Count-rate capability	• 10 to 20 cps/hydra (0.1–0.2 mC) hi-res (per 25 contiguous pixels) • 40 to 80 cps/hydra (0.4 to 0.8 mC) mid-res • 150 to 300 cps/hydra (1.5 to 3.0 mC) low-res	Accommodation of typical flux of interesting sources. Studies of accreting stellar mass black holes and neutron stars.	Essentially the count-rate per point source. When defocused, sources with *F _X_* ~ 1.5 C can be observed in the low-res mode
Ultra-Hi-Res-Array			
Ultra-Hi-Res Array parameter	Requirement	Science driver	Notes

Energy range (keV) Minimum Maximum - 1 Maximum - 2	0.2 keV 2 keV	To study faint diffuse baryons in emission, such as galactic halos	The highest energy resolution available for studies of velocities from lines up to O VIII. *R* ~ 2000.
Quantum efficiency (keV)	Area fill factor >90% Vertical QE >99% at 0.75 keV IR blocking filter throughput is largest factor affecting detection efficiency.	Maximization of counts/minimization of observation times	There are some trade-offs in filter designs - affecting whether 6 keV area is more important or area for energies below 0.6 keV.
FoV	1 × 1 arc min (3 mm × 3 mm)	To sample enough of the hot gas around galaxy halo gas	Need enough photons to measure velocities of outflows.
Pixel size (arc sec)	1 × 1 (50 *μ*m × 50 *μ*m)	To reduce the background	To get the required energy resolution!
Energy resolution up to 0.75 keV	• 0.3 eV (FWHM) (hi-res) • 0.8 eV (FWHM) (mid-res) • 2 eV (FWHM) (low-res)	Need *R* ~ 2000 to measure velocities/turbulent broadening down to ~50 km/s (outflows and thermal velocities).	Energy resolution will degrade to around 2 eV at 2 keV.
Count-rate capability	• 80 cps/1” pixels (0.8 mC) • 320 cps/pixel (3.2 mC) mid-res • 1000 cps/pixel (10 mC) low-res	- In focus, no driver. Pixel design naturally does this. - Defocused: Studies of accreting stellar mass black holes and neutron stars.	Naturally very high—will try to develop slower pixels to make it easier to read out. Count rates capability may be reduced by x-rays beyond 0.75 keV. Useful for looking at with defocused optic.

**Table 6:** This table lists the technology development milestones for the LXM for TRL-3 through TRL-6.

NASA TRL Definition	Milestones for achieving TRL-3 for the LXM (LXM)

TRL3 Analytical studies place the technology in an appropriate context and laboratory demonstrations, modeling and simulation validate analytical prediction.	Credible demonstrations must include the following for the LXM: Laboratory demonstrations of the kev technologies that are the detector and the read-out. For the detector, the operation of each of the three detector subarray pixel types should be demonstrated with form-factors (pixel size, pitch, and quantum efficiency) and performance (pitch, energy resolution, energy range) within a factor of 2 of what is needed to meet LXM requirements.
Must demonstrate a credible technology development path to the required on-orbit performance.	Specifically, they should meet the following characteristics and performance: • Multipixel detectors (hydras) demonstrated with absorber pitches <100 *μ*m, with >10 absorbers attached to each sensor, demonstrating <5 eV energy resolution (FWHM) up to 6 keV (Main Array and Enhanced Main Array). • Single-pixel detector with pitch <100 *μ*m demonstrating an energy resolution of less than 1 eV (FWHM) (Ultra-Hi-Res Array).
Demonstrations must be traceable to the on-orbit performance requirement in the operational environment.	For the read-out, the operational principle and basic performance of using microwave SQUIDs need to be demonstrated with a performance that agrees with expectation within a factor of 2. Specifically, a demonstration of the read-out should meet the following characteristics and performance: • Representative readout architecture • Degradation on intrinsic energy resolution of microcalorimeters (any type) less than a factor of 2 of what can be achieved with single-pixel read-out. • At least 30 sensors read out simultaneously on a single read-out channel. • Under the conditions of being in vacuum at realistic operating temperature and using laboratory electronics.
	Model prediction: Model predictions should show that: • Models of each of the three pixel types in the three subarrays can meet LXM performance requirements. • The electronics will be able to read-out the pixel types with properties given by detector modelling of each of the three subarrays needed by the LXM, assuming a number of read-out channels that is feasible within the cooling system and preliminary FPA designs. • A concept exists for wiring out all the pixels in the array exists using wire dimensions (pitches, thicknesses, gaps) previously demonstrated. • A concept exists for connected all the wiring of detectors to the read-out cold electronics and then to the read-out electronics at room temperature, using technologies that have previously been demonstrated. • Focal plane array design concept exists for housing the detector and cold read-out electronics, with the required number of pixels. • A cooling concept exists using existing coolers or coolers modeled and currently in development that meets all the cooling requirements of the LXM at 4 K, 1 K, and 50 mK.

NASA TRL Definition	Milestones for achieving TRL-4 for the LXM

TRL 4 A low fidelity system/component breadboard is built and operated to demonstrate basic functionality and critical test environments, and associated performance predictions are defined relative to the final operating environment.	Credible demonstrations must include the following for the LXM: A credible sensor performance budget For the detector, the operation of each of the three detector subarray pixel types should be demonstrated with form-factors (pixel size, pitch, and quantum efficiency) and performance (pitch, energy resolution, energy range) that is close (within 30%) of what is needed to meet LXM requirements. Specifically, they should meet the following characteristics and performance: • Multipixel detectors (hydras) demonstrated with absorber pitches 50 and 100 *μ*m, with 25 absorbers attached to each sensor, demonstrating <3.5 eV energy resolution (FWHM) up to 6 keV (Main Array and Enhanced Main Array).
Breadboard: A low fidelity unit that demonstrates function only, without respect to form or fit in the case of hardware, or platform in the case of software. It often uses commercial and/or ad hoc components and is not intended to provide definitive Information regarding operational performance.	• Single-pixel detector array with pixels of 50-*μ*m pitch demonstrating an energy resolution of <0.5 eV (FWHM) (Ultra-Hi-Res Array). • Demonstrate the required performance with appropriate wiring density for all pixel types.
	For the read-out, the microwave SQUIDs needs to demonstrate prototypes need to demonstrate that they are capable of reading out the three different pixel types and agree with expected performance within 30%. Specifically, a demonstration of the read-out should meet the following characteristics and performance:
Must demonstrate a credible technology development path to the required on-orbit performance. Demonstrations must be traceable to the on-orbit performance requirement in the operational environment.	• Representative readout architecture. • Demonstrate required microwave SQUID circuitry with appropriate noise and resonator widths and spacings. • Degradation on intrinsic energy resolution of microcalorimeters (any type) less than 30% of what can be achieved with single-pixel read-out. • Demonstrate basic functionality of the assumed LXM HEMT amplifier architecture. • At realistic operating temperature and using laboratory electronics. Laboratory demonstrations As a system-level bread-broad demonstration • Using arrays meeting detector and read-out subsystem level TRL-4 requirements, demonstrate the read-out of at least 100 sensors (Main Array hydras in one demonstration, UHR single pixels in a second) read out simultaneously on a single read-out channel, with energy resolution within 60% of the expected performance of the detectors. • At realistic operating temperature and using laboratory electronics.
	Model predictions: • Refined models of each of the three pixel types, based upon measured pixel parameters in the three subarrays, must meet LXM performance requirements. • The electronics will be able to read-out the pixel types with properties given by detector modeling of each of the three subarrays needed by the LXM, assuming a number of read-out channels that is feasible within the cooling System and preliminary FPA designs. • Radiation effects on flight arrays and read-out are demonstrated to not degrade performance beyond requirements for dosage expected at L2 with 5 years of operation.

NASA TRL Definition	Milestones for achieving TRLs for the LXM

TRL5 A medium fidelity system/component brassboard is built and operated to demonstrate overall performance in a simulated operational environment with realistic support elements that demonstrates overall performance in critical areas. Performance predictions are made for subsequent development phases. Brassboard: A medium fidelity functional unit that typically tries to make use of as much operational hardware/ software as possible and begins to address scaling issues associated with the operational system. It does not have the engineering pedigree in all aspects but is structured to be able to operate in simulated operational environments in order to assess the performance of criticai functions.	Credible demonstrations must include the following for LXM: For the detector subsystem, the operation of each of the three detector subarrav pixel types should be demonstrated with form-factors (pixel size, pitch, and quantum efficiency) and the performance (pitch, energy resolution, energy range) that meets the energy resolution budget requirements for the detector subarrays of the LXM. • Format, area, and wiring all pixels of a full-scale array is needed. • Pixel yield meeting requirements need to be >85%. • Protototype detector will demonstrate that performance is not degraded beyond the energy resolution budget after a radiation dose equivalent to 5 years at L2. For the read-out, the microwave SQUID read-out needs to be demonstrated that it is capable of reading out the three different pixel types and agree with expected performance within the energy resolution budget. Specifically, a demonstration of the read-out should meet the following characteristics and performance: • Representative readout architecture. • Demonstrate required microwave SQUID circuitry with appropriate noise, and resonator widths and spacings. • Degradation on intrinsic energy resolution of microcalorimeters (any type) is within the error budget allocation. • The yield of functioning read-out of all pixels meeting all noise and input slew-rate requirements is >85%. • Demonstrate exact functionality of the assumed LXM FIEMT amplifier architecture. • Prototype read-out chips will demonstrate that performance is not degraded beyond the energy resolution budget after a radiation dose equivalent to 5 years at L2. • At realistic operating temperature and using laboratory electronics. • Functional prototype electronics exist that uses flight-compatible components (or commercial versions where flight-compatible versions exist), meet the functional and expected power/mass envelopes for the LXM.
	A representative FPA needs to be demonstrated that allows the mounting of a full-scale detector and anti-coincidence detector is sufficiently large to accommodate the read-out of required number of read-out channels. Specifically, it must include: • Demonstrate that it can withstand the vibrations expected from the Lynx rocket launch. • Demonstrate wiring/cabling at the density needed for wiring out the entire array. • Demonstrate sufficient heat-sinking of the detector and read-out.
	A cooling System needs to be demonstrated that meets typical TRL-5 level cooling requirements. Specifically: • A TRL-5 4.5 K cooler needs to be demonstrated meeting the cooling requirements at 4.5 K. • A TRL-5 multistage adiabatic demagnetization cooler needs to be demonstrated meeting the cooling requirements at 50 mK and 1.0 K, generating less than 5 mW at 4.5 K.
	Laboratory demonstration of a brassboard sensor array with the following characteristics and performance: • Using arrays meeting detector and read-out subsystems meeting all TRL-5 requirements, demonstrate the read-out of required numbers of sensors (Main Array hydras in one channel, Enhanced Main Array hydras in a second, and UHR single pixels in a third) read out simultaneously on three read-out channels, with energy resolution meeting the required performance of all the detectors. • Uses a representative (TRL-5) FPA. • Assembly includes a cryogenic System and realistic support structure. • X-ray test demonstrates the feasibility of achieving Lynx performance requirements in a simulated operational environment. • At realistic operating temperature and using laboratory electronics.
	Model calculations/predictions: • Models/predictions of the full-scale LXM meets the required mass, volume and power envelopes of the LXM.

NASA TRL Definition	Milestones for achieving TRLs for the LXM

TRL 6 A high fidelity system/component prototype that adequately addresses all critical scaling issues is built and operated in a relevant environment to demonstrate operations under critical environmental conditions.	Must demonstrate using a high-fidelity scalable flight-like prototype that adequately addresses all critical scaling issues that all Lynx performance requirements are met in critical environments. A TRL-6 prototype system will be demonstrated for the LXM:
Prototype: The prototype unit demonstrates form, fit, and function at a scale deemed to be representative of the final product operating in its operational environment. A subscale test article provides fidelity sufficient to permit validation of analytical models capable of predicting the behavior of full-scale systems in an operational environment.	Prototype characteristics: • A full-size flight-like array, pixel yield meeting requirements need to be >95%. • A full set of cold flight-like read-out electronics for all channels including a full set of HEMTs at 4.5 K. The yield of functioning read-out of pixels meeting all noise and input slew-rate requirements is >95%. • A full-size fully populated mechanical prototype FPA. • Meeting all detector/readout error budget requirements. • A TRL-cooling system including a TRL-6 cryocooler and multistage ADR. • At realistic operating temperature. • Use of flight-compatible electronics that uses flight-compatible components (or commercial components where flight-compatible versions exist), meeting the functional and expected power/mass envelopes for the LXM with sufficient boards to read out half of the array. • A representative event processor operating at required rates
	Environmental testing: acoustic (blocking filter only), thermal vacuum, vibration, radiation, and x-ray testing, as appropriate, in operational environments.
